# A red-emissive antibody–AIEgen conjugate for turn-on and wash-free imaging of specific cancer cells†
†Electronic supplementary information (ESI) available: Synthesis and characterization of CSPP and CSPP-NHS, the particle size, the cell cytotoxicity test of CSPP, the parameters of mAb–CSPP and mAb–Cy3 conjugates, the PL spectra of mAb–dye conjugates *vs.* concentration, the photo-stability data, the original flow cytometric analysis data and further details of cell imaging results. CCDC 1536568. For ESI and crystallographic data in CIF or other electronic format see DOI: 10.1039/c7sc01054k


**DOI:** 10.1039/c7sc01054k

**Published:** 2017-08-17

**Authors:** Xiujuan Shi, Chris Y. Y. Yu, Huifang Su, Ryan T. K. Kwok, Meijuan Jiang, Zikai He, Jacky W. Y. Lam, Ben Zhong Tang

**Affiliations:** a Department of Chemical and Biological Engineering , Department of Chemistry , Hong Kong Branch of Chinese National Engineering Research Center for Tissue Restoration and Reconstruction , State Key Laboratory of Molecular Neuroscience , Institute of Molecular Functional Materials , Division of Life Science , The Hong Kong University of Science & Technology (HKUST) , Clear Water Bay , Kowloon , Hong Kong , China; b HKUST-Shenzhen Research Institute , No. 9 Yuexing 1st RD, South Area, Hi-Tech Park, Nanshan , Shenzhen 518057 , China; c Guangdong Innovative Research Team , SCUT-HKUST Joint Research Laboratory , State Key Laboratory of Luminescent Materials and Devices , South China University of Technology , Guangzhou 510640 , China

## Abstract

An antibody–AIEgen conjugate is designed and developed as a “turn-on” fluorescent probe for wash-free specific cancer cell imaging. The cetuximab-conjugated AIEgen shows red fluorescence only when it is internalized and accumulated in cancer cells with overexpressed epidermal growth factor receptor through endocytosis. The probe first lights up the lysosomes. After hydrolysis, its residue is accumulated in mitochondria, making them highly emissive with a long cell retention time. Compared with conventional “always-on” fluorescent probes, the antibody–AIEgen conjugate exhibits a very good image contrast during wash-free cancer cell imaging and less interference from normal cells. To the best of our knowledge, this is the first time “turn-on” antibody–AIEgen conjugates have been reported. This new strategy can be further extended to many proteins and water-soluble AIEgens, and many of their potential applications such as real-time tracking of cell dynamics and cancer theranostics will be explored. The present work is expected to inspire more marvellous research in the fields of AIE and cancer imaging.

## Abstract 2

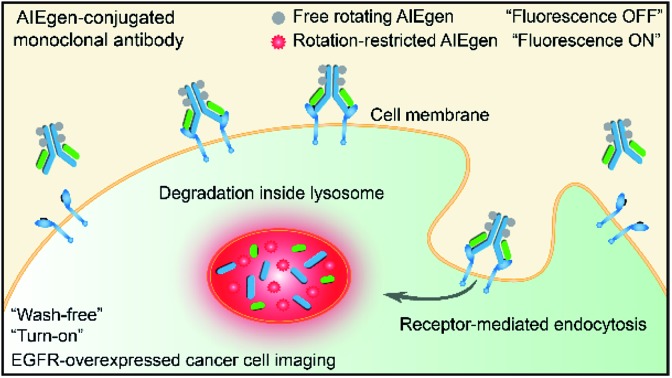
For the first time, an AIEgen-conjugated monoclonal antibody is designed for “turn-on” and “wash-free” imaging of EGFR-overexpressed cancer cells.

## Introduction

Medical imaging technologies, including radionuclide imaging, X-ray computed tomography, magnetic resonance imaging, optical imaging *etc.*, are very important in medical applications, particularly in the early diagnosis and treatment of diseases as well as for basic pharmaceutical development.^1^,^2^ Molecular imaging using biomarkers to image and monitor the spatiotemporal distribution of targets or biological processes has significant economic and medical impacts due to the earlier and more precise diagnosis.^3^ One of the most challenging aspects of designing a probe for medical imaging is to optimize the target-to-background ratio (TBR). In principle, images with high contrast, sensitivity, and specificity can be achieved by maximizing the signal from the targets and/or minimizing the signal from the background.^4^ Conjugating a monoclonal antibody (mAb) to a probe has been proven to be a good strategy for designing specific molecular imaging probes, due to the excellent target specificity of antibodies.^5^ mAb possesses the pharmacokinetic features of a relatively prolonged circulating half-life and slow clearance from the body.^5^ Conventional “always-on” antibody-conjugated probes, such as iodine, gadolinium, radioisotopes, fluorescent dyes, and quantum dots, suffer from high non-specific background signals from the blood pool and normal tissues, which results in a very low TBR and a poor image contrast.^6^,^7^ However, antibody-conjugated probes with “turn-on” features generate a signal only upon binding to targets. By combining an antibody with a turn-on fluorescent dye, maximum target signal and minimum background signal could be achieved, and thus a high TBR and high image contrast result.

Fluorescence-based imaging techniques exhibit the advantages of high sensitivity, high resolution, dynamic imaging, low cost, and convenient portability, and are one of the easiest ways to develop “turn-on” probes.^6^,^8^,^9^ Specifically, fluorescent targeting agents have been widely adopted for early cancer diagnosis and fluorescence-guided surgery to determine and visualize tumour margins, thus improving the surgical outcome for cancer patients.^5^,^10^ There are several strategies for designing fluorescent “turn-on” antibody probes, such as fluorescence resonance energy transfer (FRET), H-dimer formation, and photo-induced electron transfer (PET).^6^,^8^ For example, the fluorescence of probes is activated by lysosomal conditions in specific cells including low pH, oxidation, unfolding, catabolism, or protein cleavage by lysosomal enzymes.^6^ However, not all fluorophores are able to form an H-dimer and their emissions are hard to fully extinguish upon conjugation with an antibody. In order to attain the emission quenching, multiple fluorescent molecules are labelled in one antibody. However, such a high labelling ratio probably interferes with the binding affinity. In addition, the nonspecific adsorbed dyes on antibody and dye molecules that are released after catabolism may return to blood circulation, generating false-positive signals.^11^ Besides, the development of antibody probes with FRET is complicated as it requires a well matching and precise distance between the donor and the acceptor. Moreover, the design and synthesis of PET dyes that can conjugate with an antibody is also a difficult task. Thus, a simple design of fluorescent molecules with improved properties remains highly desirable for developing fluorescent “turn-on” antibody probes.

**Scheme 1:**
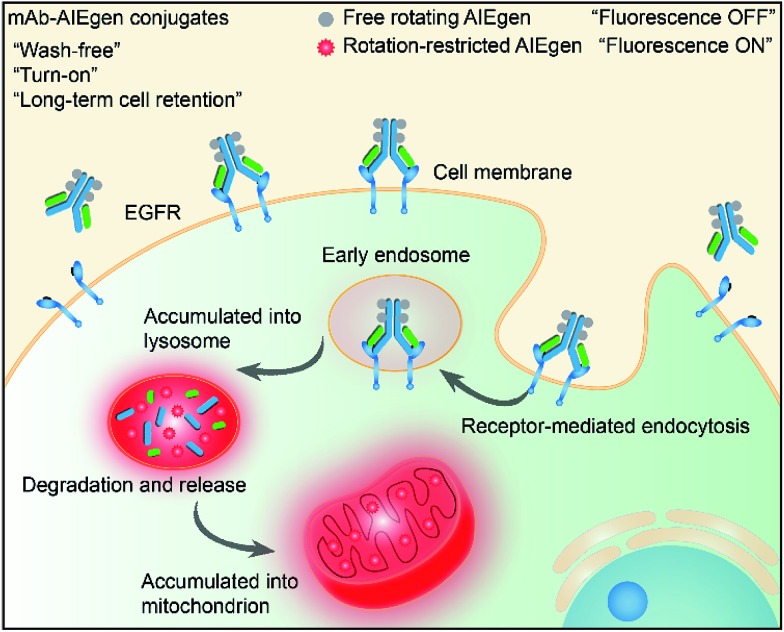
Schematic representation of the “turn-on” process of specific cancer cells by mAb–AIEgen conjugates.

Recently, luminogens with aggregation-induced emission (AIE) characteristics have attracted intense research attention. In contrast to fluorophores with aggregation-caused quenching (ACQ) effects, AIE luminogens (AIEgens) offer only weak fluorescence when they are molecularly dissolved in good solvents. Their emissions, however, are greatly amplified in the aggregated state or in a confined environment, owing to the restriction of intramolecular motion (RIM).^12^,^13^ Such unique features of AIEgens provide a powerful rationale to develop both “always-on” and “turn-on” imaging tools. AIE nanoparticles or dots (“always-on” probes) exhibit advantages in bioimaging over conventional organic dyes and quantum dots, such as high brightness, excellent biocompatibility, being free of random blinking, and having strong photobleaching resistance.^14^–^17^ Additionally, many “turn-on” AIE bioprobes have been designed for detecting specific biomolecules and bacteria.^18^–^25^ In particular, AIEgens decorated with hydrophilic peptides have been reported as “light-up” probes for cancer imaging with high contrast.^26^–^31^ The water-soluble peptide chain enables the AIEgen to be molecularly dissolved in aqueous media to extinguish the emission. However, the probe lights up due to RIM upon specific recognition.^31^,^32^ Unfortunately, hydrophilic peptides generally have a short half-life due to rapid degradation by proteases and rapid clearance from circulation. Coupled with their conformational flexibility, they generally show low selectivity.^33^ In comparison to peptides, mAb possesses a higher specificity and greater diversity, and has been widely applied in clinical and pre-clinical studies.^5^,^8^,^34^,^35^ Although the biological applications of AIEgens have flourished in recent years, no “turn-on” antibody–AIEgen conjugate has been prepared so far. This is possibly because antibodies are inconvenient to handle as they require specific pH and temperature conditions to function. Meanwhile, almost all of the reported AIEgens are hydrophobic. This makes it easy for them to form aggregates in aqueous solution, and thus they barely react with proteins or even destroy the bioactivity of proteins during conjugation. Although some water-soluble AIEgens have been developed, their excitation and emission wavelengths are short, making them suffer from strong interference from the cell autofluorescence and short penetration depth.^4^ Very recently, we published work on the synthesis of mAb-decorated AIE dots that can image specific cancer cells.^15^ However, these cannot be used for “wash-free” and “turn-on” imaging. Inspired by the successful research in developing water-soluble AIEgens, in this contribution, we designed a new water-soluble and red-emissive AIEgen (CSPP-NHS) and utilized it to prepare a “turn-on” antibody-conjugated AIEgen probe. As a proof of concept, cetuximab, an FDA approved mAb exhibiting a high affinity to human epidermal growth factor receptor (EGFR),^34^ was used to conjugate with CSPP-NHS to afford a mAb–CSPP conjugate. EGFR is abnormally amplified in a variety of tumors and has been identified as an important target in cancers.^36^ The imaging process is illustrated in Scheme 1; the mAb–CSPP conjugate emitted very weakly in aqueous solution due to the non-radiative decay of the excited state caused by the free intramolecular rotation, while it became highly emissive after internalization induced by EGFR mediated endocytosis. The mAb–CSPP first turned on in the lysosome, where its hydrolysis was promoted. The resulting cationic AIE metabolites then moved and accumulated in mitochondria, imparting them with strong light emission and long-term cell retention. It is the excellent combination of the novel AIE effect with the characteristics of biological environments in a positively cooperative manner that generates the “turn-on” protein probe.

## Results and discussion

### Design and optical properties of CSPP

**Fig. 1:**
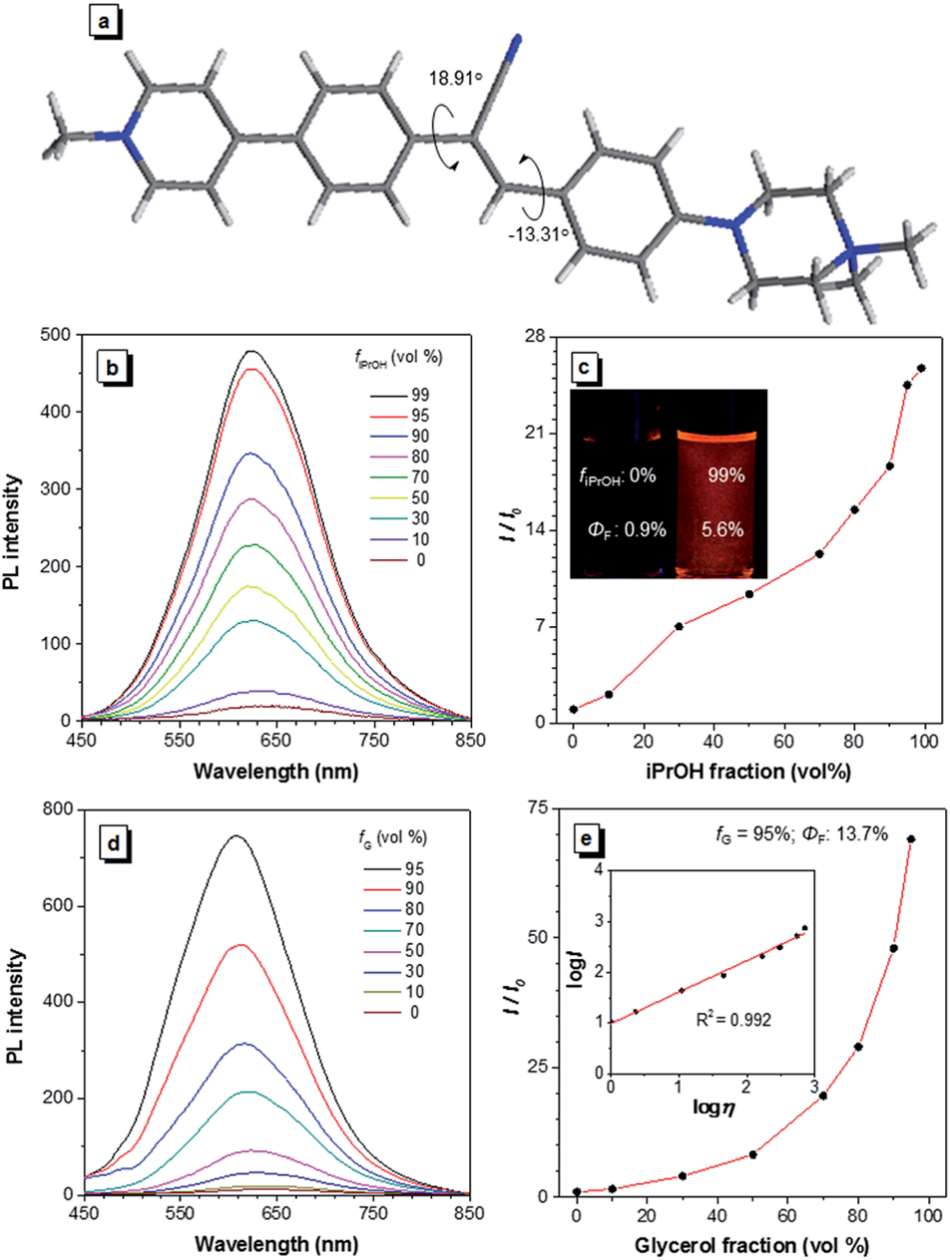
(a) The crystal structure of CSPP. (b) The PL spectra of CSPP in water/iPrOH mixtures with different iPrOH fractions (*f*_iPrOH_). (c) A plot of relative intensity (*I*/*I*_0_) at 640 nm *versus f*_iPrOH_, where *I*_0_ was the PL intensity in pure water. Inset: photographs of CSPP in water/iPrOH mixtures with 0% and 99% iPrOH fractions taken under 365 nm UV irradiation. (d) The PL spectra of CSPP in water/glycerol mixtures with different glycerol fractions (*f*_G_). (e) A plot of *I*/*I*_0_ at 620 nm *versus f*_G_, where *I*_0_ was the PL intensity in pure water. Inset: plot of log *I* against log *η*, where *η* was the solution viscosity. Concentration: 10 μM, *λ*_ex_: 400 nm.

A water-soluble AIEgen with long absorption and emission wavelength is highly desirable for antibody labelling because it can avoid the use of organic solvents during the conjugation and minimize the cell autofluorescence during imaging. α-Cyanostilbene is an AIEgen with a simple molecular structure and benefits from ease of functionalization, and it was therefore selected as the AIE core of CSPP (Fig. 1a) and CSPP-NHS. The synthetic details, NMR spectra, and MS spectra of CSPP and CSPP-NHS are described and given in Fig. S1–S7 in the ESI.† Electron-deficient pyridinium salt and electron-rich piperazine moieties were decorated on both sides of α-cyanostilbene. This built a donor–acceptor structure, which helped to shift the absorption and emission of the AIEgen to a redder region. The hydrophilic units endowed them with water solubility. CSPP was used as the model compound for investigation of the properties. It was soluble in water and its aqueous solution emitted weak red photoluminescence (PL) at 640 nm with a fluorescence quantum yield (*Φ*_F_) of 0.9% (Fig. 1b). With the increment of the iPrOH fraction (*f*_iPrOH_) in the CSPP aqueous solution, the emission gradually intensified. At 99% *f*_iPrOH_, a 25-fold enhancement of PL intensity (*Φ*_F_ = 5.6%) was recorded, which is due to the aggregate formation and the AIE effect of CSPP (Fig. 1c and S8, see ESI†). The PL of CSPP also showed an exponential growth with increasing solution viscosity. There was a 68-fold enhancement of PL intensity (*Φ*_F_ = 13.7%) at 620 nm in a mixture with a 95% glycerol fraction as compared with that in water. A linear relationship between log *I* and log *η* was found, where *I* is the PL intensity and *η* is the solution viscosity (Fig. 1d and e). To further understand the AIE phenomenon of the CSPP molecule, we analysed its single crystal structure (Fig. 1a). The molecule adopted a slightly twisted conformation with dihedral angles of 18.91° and –13.31°. The phenyl rings adjacent to the acrylonitrile group could rotate freely in water, which consumed the energy of the excitons non-radiatively. The RIM process was activated in the aggregated state or in a highly viscous solution, thus endowing the dye with strong light emission.

Before applying CSPP in antibody labelling and cell imaging, we first evaluated its cytotoxicity *via* CCK-8 assay. The cell viability of HCC827 and NCI-H23 cells were above 90% and 81%, respectively, at CSPP concentrations of up to 50 μM, demonstrating its low cytotoxicity (Fig. S9, see ESI†). It is noteworthy that no aggregates of CSPP were formed, even at a high solution concentration of 2 mM, but aggregates appeared at 3 mM, as measured by DLS (Fig. S10, see ESI†). This suggests that CSPP possesses a very good water solubility, thus making it favourable for protein conjugation in aqueous solution.

### Characterization of mAb–CSPP conjugates

**Fig. 2:**
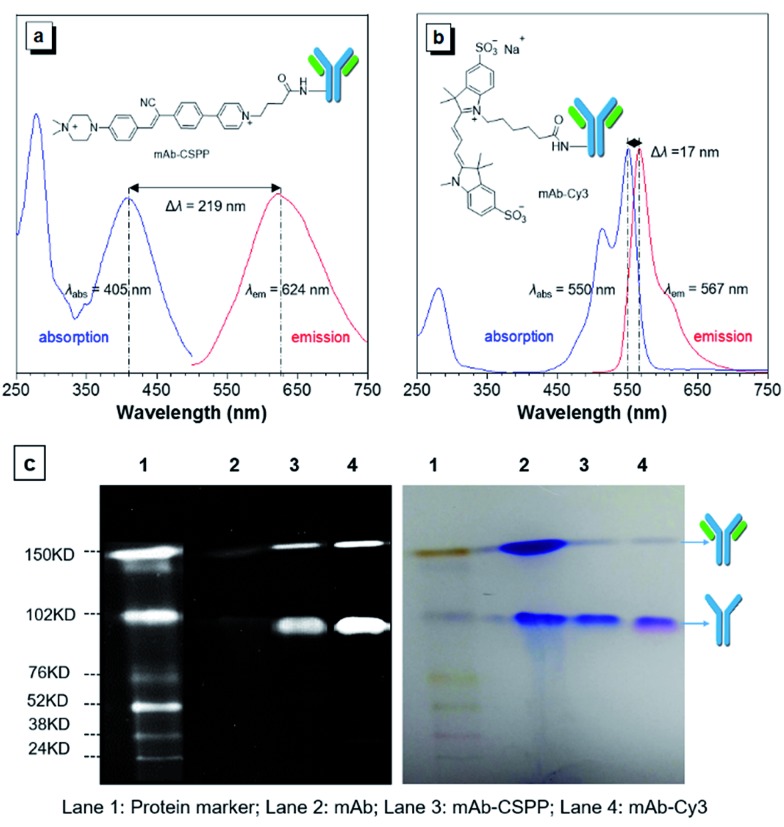
Absorption and emission spectra of (a) mAb–CSPP and (b) mAb–Cy3 conjugates. Conjugate concentration: 0.2 mg mL^–1^. (c) SDS-PAGE of monoclonal antibody cetuximab (mAb), and mAb–CSPP and mAb–Cy3 conjugates. Left: the protein marker was excited by the Cy5 (635 nm) and Cy3 (532 nm) channels, mAb–CSPP was excited by UV channel and the mAb–Cy3 was excited at Cy3 channel. Right: All protein bands were stained with Coomassie Brilliant Blue showing the intact antibody and the partly reduced antibody with two heavy chains left.

The *N*-hydroxy-succinimidyl (NHS) ester group has been popularly adopted for protein–dye conjugation. It can react with lysine residues of antibodies to form amide bonds.^37^ In this work, NHS-functionalized CSPP (CSPP-NHS) was prepared and conjugated with cetuximab to afford mAb–CSPP (Fig. 2a). For a comparison, the “always-on” sulfo-Cy3-NHS ester was also used to conjugate with mAb to give mAb–Cy3 (Fig. 2b). The success in antibody conjugation was proved using SDS-PAGE. As shown in Fig. 2c, the mAb–CSPP and mAb–Cy3 conjugates exhibited bright fluorescence at the corresponding antibody bands when illuminated by light from the UV channel and the Cy3 channel of 532 nm, respectively. The essential parameters of the mAb–dye conjugates are listed in Table S1 in the ESI.† The dye–protein ratio was determined to be ∼3 using UV spectroscopy. mAb–CSPP showed an absorption maximum at 405 nm with a molar absorptivity of 26 800 L mol^–1^ cm^–1^ and emitted weakly at 624 nm, giving a large Stokes shift of 219 nm. In contrast, mAb–Cy3 showed only a small Stokes shift of 17 nm. The large Stokes shift of mAb–CSPP means that it can significantly avoid self-absorption, making the choice of emission filters easy. In addition to the difference in Stokes shift, the mAb–Cy3 conjugates exhibited emission self-quenching at solution concentration of above 0.22 mg mL^–1^. On the contrary, the fluorescence of the mAb–CSPP conjugates increased along with concentration, up to 1.5 mg mL^–1^ (Fig. S11, see ESI†). Since the fluorescence of mAb–CSPP is positively correlated with its concentration, a more correct estimation on the imaging results can be carried out. However, mAb–Cy3 may give a false signal due to the concentration-induced emission quenching.^38^

Cetuximab has been widely applied for targeting non-small cell lung cancer (NSCLC) with overexpressed EGFR.^39^ Among the different NSCLC cell lines, our group has sieved HCC827 cells with the highest level of EGFR expression and NCI-H23 cells with the lowest expression based on the results of immunoblotting analysis.^15^ These two cell lines were used to evaluate the bioactivity of the mAb–CSPP conjugates that underwent covalent conjugation and several purification processes. As shown in Fig. S12 in the ESI,† no fluorescence was detected in NCI-H23 cells after being incubated with mAb–CSPP for 24 h, while bright fluorescence was observed in HCC827 cells, indicating the good bioactivity and selectivity of mAb–CSPP to EGFR-overexpressed cancer cells.

Photostability is a pivotal parameter for fluorescence imaging. Herein, we measured the photostability of mAb–CSPP and mAb–Cy3 by continuous scanning the HCC827 cells stained by them (Fig. S13, see ESI†). The results showed that the photostability of mAb–CSPP was comparable to that of mAb–Cy3.

### Comparison of “turn-on” and “always-on” probes in terms of background fluorescence

**Fig. 3:**
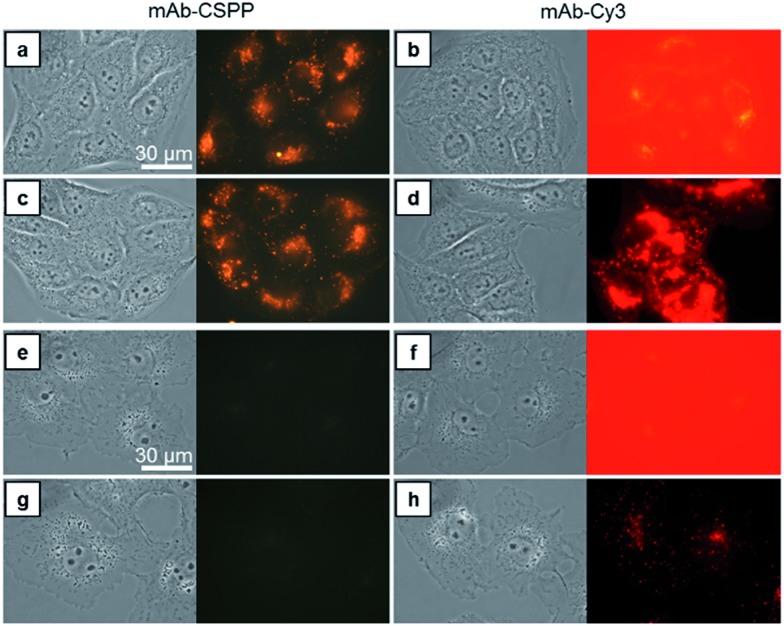
Bright-field and fluorescent images of (a–d) HCC827 cancer cells and (e–h) COS-7 normal cells incubated with 10 μg mL^–1^ of mAb–CSPP and mAb–Cy3 conjugates for 12 h. Images were taken (a, b, e and f) in the probe medium without washing and (c, d, g and h) in the medium without probe after washing. Conditions: for mAb–Cy3, *λ*_ex_ = 510–550 nm, dichroic mirror = 570 nm; for mAb–CSPP, *λ*_ex_ = 400–440 nm, dichroic mirror = 455 nm. Exposure time = 1 s; scale bar = 30 μm.

Compared with CSPP in PBS buffer, the mAb–CSPP conjugates showed a 5.4-fold increase of PL intensity measured at a high excitation slit width (Fig. S14, see ESI†). The emission, however, was still weak, indicating that the intramolecular motion of CSPP was not fully restricted on the antibody surface. Nevertheless, a low background signal should be envisioned. On the other hand, using the same excitation slit width, mAb–Cy3 conjugates were highly emissive at a protein concentration of 0.1 mg mL^–1^ (Fig. S15, see ESI†). Then, these antibody probes were applied to do wash-free imaging of both cancer and normal cells. As expected, the “always-on” probe showed a high background fluorescence from the probe medium (Fig. 3b), even submerging the signals from the cancer cells (Fig. 3d). In contrast, mAb–CSPP showed almost no background fluorescence from the probe medium (Fig. 3a) and only emitted in HCC827 cells with overexpressed EGFR (Fig. 3a and c). It is well-known that EGFR is widely expressed in many cell types, including epithelial and mesenchymal lineages because it plays an essential role in regulating normal cell signaling.^40^–^42^ However, its overexpression accounts for the pathogenesis and progression of cancer cells.^43^ Accordingly, we also checked the background fluorescence from normal cells, which were imaged with both wide-field fluorescence microscopy and laser scanning confocal microscopy (LSCM). For wash-free imaging, the mAb–Cy3 probe showed a very low image contrast between HCC827 (Fig. 3b) and COS-7 (African green monkey fibroblast-like cells, Fig. 3f) due to the high fluorescence background from the unbound antibody probes. On the contrary, the mAb–CSPP probe showed a high image contrast because of the very low background signal from unbound antibody probes (Fig. 3a and e). In addition, fluorescence was observed from COS-7 normal cells with low level of EGFR expression^40^ after incubation with mAb–Cy3 for 12 h and PBS washing (Fig. 3h). In contrast, almost no emission was shown in COS-7 cells stained by mAb–CSPP (Fig. 3e and g). The background signal from other normal cells was also checked. The results showed that the wash-free imaging of EGFR-negative HEK-293 normal cells (human embryonic kidney epithelial cells)^44^ incubated with mAb–Cy3 exhibited high background emission from the unbound mAb–Cy3 probe (Fig. S16b, see ESI†). Furthermore, the HEK-293 cells incubated by mAb–Cy3 showed a little fluorescence signal even after washing, which probably resulted from the small amount of nonspecific adsorption of the antibody. In contrast, undetectable fluorescence was found from HEK-293 normal cells stained by mAb–CSPP for 12 h with or without washing (Fig. S16a and c, see ESI†). In addition, other normal cells NIH 3T3 (mouse embryonic fibroblast cells) and MDCK.2 (Madin–Darby canine kidney epithelial cells) with scarce EGFR expression^41^,^42^ exhibited almost no fluorescence after incubation with mAb–CSPP for 12 h (Fig. S17a and b, see ESI†). However, the background fluorescence was still detectable from NIH 3T3 and MDCK.2 cells stained by mAb–Cy3 (Fig. S17c and d, see ESI†).

In short, mAb–Cy3, being an “always-on” probe, exhibited high background fluorescence from both the probe medium and normal cells. In contrast, mAb–CSPP showed almost no background fluorescence. This helped to increase the TBR and improve the image contrast, endowing mAb–CSPP with the potential to differentiate tumors from normal tissues. It can be envisioned that antibody probes fabricated from the conventional “always-on” luminogens will always give a high background signal coming from the unbound probe, the catabolites, and the drop-off of non-covalently adsorbed dyes on mAb.^11^ In contrast, the mAb–AIEgen probe will give a low background signal due to the free rotation of the AIEgens, which provides a non-radiative pathway for the excitons to relax.

### Turn-on process of HCC827 cells during wash-free imaging

**Fig. 4:**
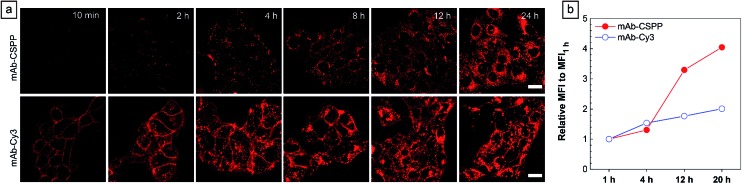
(a) LSCM images of HCC827 cells incubated with 10 μg mL^–1^ mAb–CSPP conjugates at different incubation times without PBS washing and 10 μg mL^–1^ mAb–Cy3 at different incubation times after PBS washing. Conditions: for mAb–Cy3, *λ*_ex_ = 560 nm, emission filter = 563–700 nm. For mAb–CSPP, *λ*_ex_ = 405 nm, emission filter = 550–700 nm. Scale bar: 20 μm. (b) Flow cytometric analysis of HCC827 cells after incubation with mAb–CSPP and mAb–Cy3 conjugates at different time intervals. The plot shows the relative fluorescence intensity (MFI/MFI_1 h_) with probe incubation time, where MFI_1 h_ is the mean fluorescence intensity after probe incubation for 1 h.

**Fig. 5:**
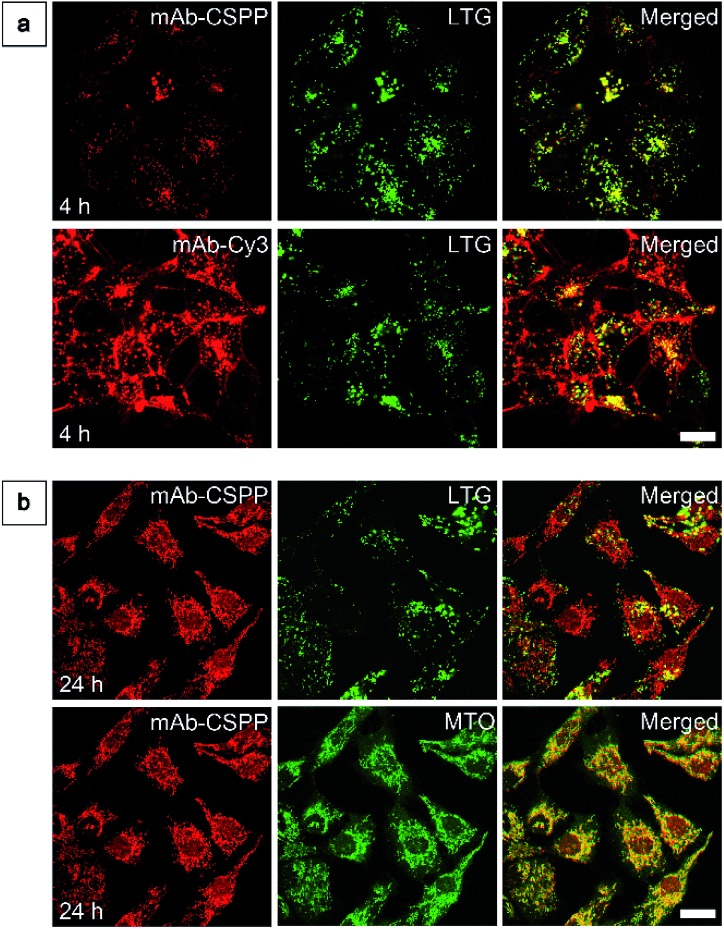
(a) LSCM images of HCC827 cells stained with 10 μg mL^–1^ mAb–CSPP or mAb–Cy3 conjugates for 4 h and then co-stained with LysoTracker Green (LTG) for 5 min. (b) LSCM images of HCC827 cells stained with 10 μg mL^–1^ mAb–CSPP for 24 h, followed by staining with LTG/Mito-Tracker Orange (MTO). Conditions: for mAb–Cy3, *λ*_ex_ = 560 nm, emission filter = 563–700 nm. For mAb–CSPP, *λ*_ex_ = 405 nm, emission filter = 550–700 nm. For LTR, *λ*_ex_ = 488 nm, emission filter = 495–535 nm. For MTO, *λ*_ex_ = 560 nm, emission filter = 565–585 nm. Scale bar: 20 μm.

To show the “turn-on” process of mAb–CSPP conjugates in the targeted HCC827 cells, serial LSCM images at selected time points were obtained in real-time without washing. Like many commercial fluorescent probes, mAb–Cy3 showed strong light emission in the probe medium and required washing with PBS at a selected incubation time before acquiring the LSCM images. As the washing steps will take extra time, measurement of biological processes on a short time-scale becomes impossible. Herein, we ignored the time-based limitation to real-time cell imaging using mAb–Cy3. It is well-known that cetuximab, after binding with EGFR, produces receptor dimerization, undergoes internalization into endosomal and lysosomal vesicles and induces degradation of the internalized antibody–receptor complex and down-regulation of the receptor.^45^–^50^ Therefore the two antibody probes will track the fate of cetuximab after binding with EGFR. As shown in Fig. 4a, HCC827 cells incubated with mAb–Cy3 revealed fluorescence on the cell membrane in the first 10 min. Bright fluorescence spots appeared inside the cells after 2 h, indicating the endosomal–lysosomal uptake of the probe.^51^ Furthermore, after 2 h the mAb–Cy3 showed strong fluorescence both on the cell membrane and inside the cells without spatial distinguishability as proved by the co-staining with LysoTracker Green at 4 h incubation (Fig. 5a). In contrast, no fluorescence was observed on the cell membrane incubated with mAb–CSPP at 10 min (Fig. 4a). After binding with EGFR, cetuximab is slowly internalized early into the endosome and transferred to the lysosomes for degradation.^45^–^47^ After 2 h incubation, only a few punctate fluorescent spots were observed inside the cells. Then, the cells became brighter along with the incubation time because more mAb–CSPP conjugates were endocytosed. It has been reported that the internalization of antibody–receptor complexes is minimal in the first 2 h.^7^,^52^ This is consistent with what was observed here. Other activatable antibody probes also showed a time-dependent fluorescence increase.^7^,^11^,^52^,^53^ At 4 h, the fluorescence of mAb–CSPP co-localized well with LysoTracker Green with a Pearson correlation coefficient of 0.87 (Fig. 5a). However, it was surprising to find that the fluorescence of mAb–CSPP existed not only on lysosomes but also on mitochondria at 24 h, as revealed by the good co-localization with LysoTracker Green and MitoTracker Orange (Fig. 5b). In particular, mAb–CSPP was mainly located in the lysosome at 8 h, but began to migrate to other organelles at 12 h as its fluorescence did not overlap well with LysoTracker Green (Fig. S18, see the ESI†). In order to gain a better understanding, we stained HCC827 cells with 5 μM of CSPP directly for 8 h. The co-staining experiment with MitoTracker Green showed a good overlap with CSPP with a Pearson correlation coefficient of 0.80. This indicated that the cationic CSPP itself can specifically target mitochondria (Fig. S19, see the ESI†). It was inferred that the mAb–CSPP conjugates in the lysosome were hydrolysed due to the harsh environments, releasing the cationic CSPP catabolites, which were likely to accumulate in mitochondria as driven by the high membrane potential of mitochondria. The “turn-on” process was also checked quantitatively *via* flow cytometry (Fig. 4b and S20, see the ESI†). Compared with the mean fluorescence intensity (MFI) after probe incubation for 1 h, the MFI increased about 0.30-fold, 2.30-folds and 3.05-fold after HCC827 cells were incubated with mAb–CSPP for 4 h, 12 h, and 20 h, respectively. In contrast, the MFI increased only a little for cells incubated with mAb–Cy3 for longer time (Fig. 4b) due to its “always-on” property. Hence, mAb–CSPP incubated HCC827 cells showed a time-dependent internalization of mAb–CSPP, where cells were slowly lighting up with time. It was indicated that the “turn-on” process of the mAb–CSPP probe to HCC827 cells was responsive to the endocytosis process, and highly related to its location inside cells.

### Endocytosis-induced emission

**Fig. 6:**
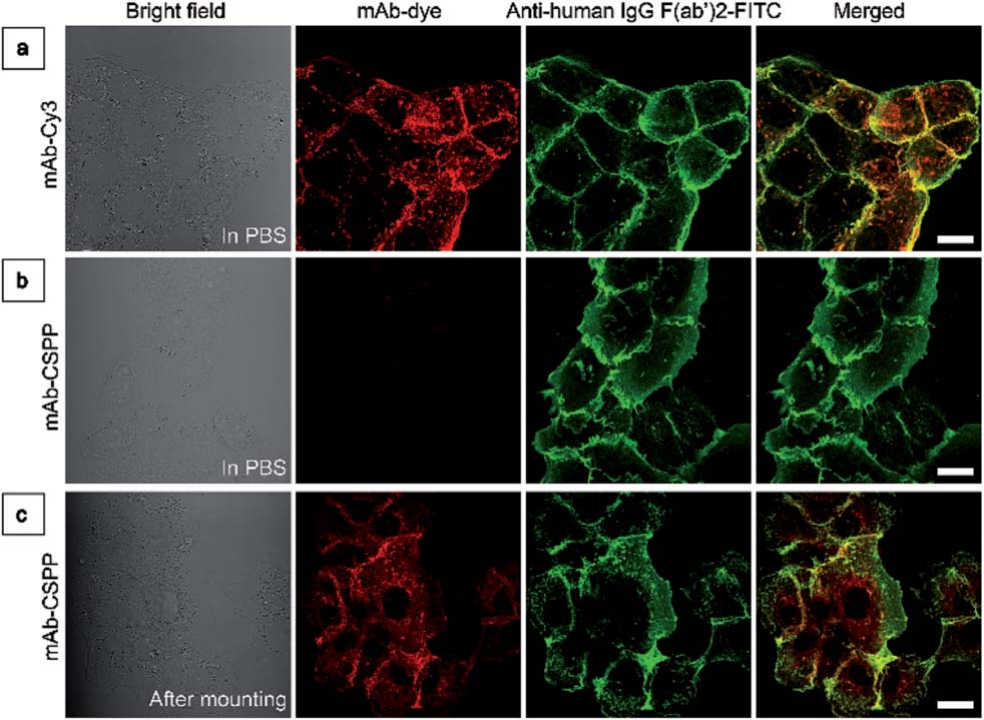
(a) LSCM images of HCC827 cells co-stained with mAb–Cy3 and anti-human IgG F(ab′)2-FITC taken in PBS. (b and c) LSCM images of HCC827 cells co-stained with mAb–CSPP and anti-human IgG F(ab′)2-FITC taken (b) in PBS and (c) after cell mounting with Fluoromount. Conditions: for mAb–Cy3, *λ*_ex_: 560 nm; filter = 563–700 nm. For FITC, *λ*_ex_ = 488 nm, filter = 495–535 nm. For mAb–CSPP, *λ*_ex_ = 405 nm, filter = 550–700 nm. Scale bar: 20 μm.

To investigate the “turn-on” mechanism of the mAb–CSPP probe, several questions should be answered. First, why is mAb–AIEgen not emissive on the cell membrane? A set of immunofluorescence experiments were performed in order to figure out whether mAb–CSPP was located on cell membrane or not. After probe incubation for 25 min, the cells were fixed, permeablized and blocked. Afterwards, they were incubated with goat F(ab′)2 anti-human IgG F(ab′)2 (FITC), which could specifically recognize human constant subunits presented on the IgG F(ab′)2 of cetuximab. As shown in Fig. 6a, the pseudo-green colour of goat F(ab′)2 was overlaid very well with the pseudo-red colour of mAb–Cy3 on the cell membrane. For cells incubated with the mAb–CSPP probe, only the pseudo-green colour appeared (Fig. 6b). This indicated that mAb–CSPP indeed docked on cell membrane initially, but it was in the dark state. Unexpectedly, the emission of mAb–CSPP on the cell membrane turned on and was co-localized with the pseudo-green colour of goat F(ab′)2 after the cells were mounted with mounting medium (Fig. 6c). When the cetuximab–CSPP conjugates interacted with EGFR on the cell membrane, the intramolecular motion of CSPP was partially constrained but this was still not enough to give a detectable emission. Such behaviour is different from peptide-decorated AIEgens as reported by Liu *et al.* and Ding *et al.* The small peptide-decorated AIEgens can reach into the cavity of the protein receptors on the cell membrane^27^,^32^ or form assemblies/aggregates in protein clusters on the cell membrane.^54^ This activated the RIM process and thus enhanced the fluorescence of the probe. However, the recognition between mAb and the receptors did not help much to restrict the intramolecular motion of the AIEgen because the small molecular AIEgen might not necessarily conjugate at the recognition sites of mAb.^55^ Thus, the dye molecule could freely rotate in the aqueous environment. When using mounting medium to solidify the cell sample (Fig. 6c), the intramolecular motion of CSPP was largely restricted, leading to high fluorescence emission. From this perspective, we speculate that any AIEgen capable of labelling proteins are able to be used in immunocytochemistry, where the fluorescence of the dye molecule will turn on after cell mounting.

**Fig. 7:**
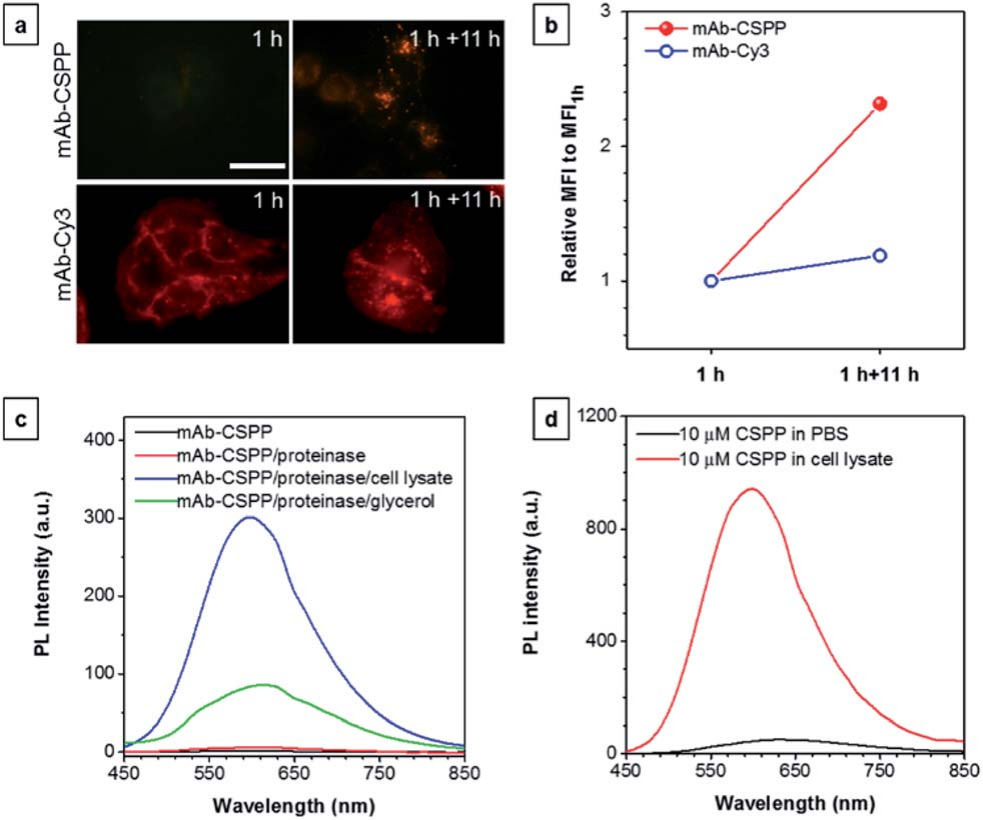
(a) Fluorescence images of HCC827 cells incubated with 10 μg mL^–1^ of mAb–CSPP and mAb–Cy3 conjugates for 1 h and the fluorescence images of dye-stained cells following washing with PBS and further incubation in a fresh medium for 11 h. Conditions: for mAb–Cy3, *λ*_ex_ = 510–550 nm, dichroic mirror = 570 nm. For mAb–CSPP, *λ*_ex_ = 400–440 nm, dichroic mirror = 455 nm. Exposure time: 2 s. Scale bar: 30 μm. (b) Flow cytometric analysis of HCC827 cells after incubation with mAb–CSPP and mAb–Cy3 conjugates for 1 h, followed by incubation in a fresh medium without the antibody probe for 11 h. The plot shows the relative fluorescence intensity (MFI/MFI_1 h_), where MFI_1 h_ is the mean fluorescence intensity after probe incubation for 1 h. Conditions: for mAb–Cy3, *λ*_ex_ = 561 nm, detection with bandpass filter = 583 ± 7.5 nm. For mAb–CSPP, *λ*_ex_ = 405 nm, detection with bandpass filter = 610 ± 10 nm. (c) The PL intensity change of the digested mAb–CSPP conjugates catalyzed by Proteinase K upon addition of cell lysate and 50% glycerol. The concentration of mAb–CSPP used for the fluorescence measurement was 0.2 mg mL^–1^. (d) The PL intensity change of 10 μM of CSPP induced by cell lysate.

To further prove the fluorescence responsiveness of mAb–CSPP to endocytosis, we compared the fluorescence of HCC827 cells incubated with mAb–CSPP for 1 h with that of cells incubated with mAb–CSPP for 1 h followed by endocytosis in a fresh culture medium for 11 h to avoid the influence of more antibody binding from the culture medium. After 1 h of incubation, mAb–Cy3 was found to be mainly located on the cell membrane (Fig. 7a). The cell membrane of HCC827 cells incubated with mAb–CSPP for 1 h was non-emissive (Fig. 7a), but emission was observed inside cells after 11 hours of endocytosis (Fig. 7a). Additionally, the MFI increased 1.4-fold after 11 hours of endocytosis compared with the beginning fluorescence of HCC827 cells incubated with mAb–CSPP for 1 h, while almost no increase of MFI was observed for mAb–Cy3 as suggested by the results from flow cytometer (Fig. 7b and S21, see the ESI†). These experiments demonstrated that the fluorescence of mAb–CSPP was only turned on after receptor-mediated endocytosis. But why is the fluorescence turned on inside the lysosome? We assumed that the lysosome environment and antibody degradation inside the lysosome were related to the emission enhancement of mAb–CSPP. In order to prove this hypothesis, Proteinase K was used to digest the mAb–CSPP conjugates according to a reported method with a little change.^56^ After antibody digestion, the released CSPP residues will interact with the surrounding environment freely. The results showed that the intact mAb–AIEgen and the mixture of degraded mAb–AIEgen and Proteinase K emitted very weakly (Fig. 7c). To simulate the environment of the lysosome, cell lysate was added. It was found that the fluorescence of degraded mAb–AIEgen increased about 49 times (Fig. 7c). Since the CSPP molecule has good water-solubility, the great fluorescence increase may be attributed to the electrostatic interactions of positively charged CSPP with biomolecules inside cells (Fig. 7d). Considering that the viscosity of lysosomes (130 ± 20 to 175 ± 20 cP)^57^ may also play a role, we added glycerol into the degraded mAb–CSPP solution with a volume of 50% glycerol to acquire a viscosity approximately equal to that of the lysosome. The fluorescence increased 13 times (Fig. 7c). Therefore, the reason for lighting up the lysosome was in part due to the viscosity effect, and mainly due to the degradation of the antibody, which allows the free interactions of AIEgens with the surrounding environment leading to the large restriction of the intramolecular rotation of AIEgens and greatly enhanced fluorescence emission.

Based on the “turn-on” mechanism, the early endocytosis into endosomes did not help much in terms of the fluorescence enhancement at the beginning of cell incubation with mAb–CSPP probe, because the antibody was intact and also the surrounding environment was highly hydrophilic, according to the work reported by Chen *et al.*^58^ After antibody degradation in the lysosome, the cationic CSPP catabolites were slowly released from the lysosome and accumulated in mitochondria. The emission of CSPP was also turned on in mitochondria probably by the interactions of CSPP with the mitochondrial matrix.

After understanding the “turn-on” process of the mAb–CSPP conjugates in HCC827 cells with EGFR overexpression, it was not hard to understand why normal cells incubated with mAb–CSPP showed very low background (Fig. 3g, S16c, S17a and b, see ESI†). In general, the fluorescence of mAb–CSPP is highly dependent on where it is and its abundance in cells. When the probe was on the cell membrane or at the early stage of endocytosis, the emission was kept in the dark state. A small amount of endocytosed probes in normal cells would not give enough emission. Cancer cells with overexpressed EGFR have a higher amount of EGFR and faster metabolism than normal cells. So the fluorescence in cancer cells would increase a lot after the mAb–CSPP conjugates were internalized or catabolized, thus increasing the TBR.

### The *in vitro* stability of the mAb–CSPP conjugates

**Fig. 8:**
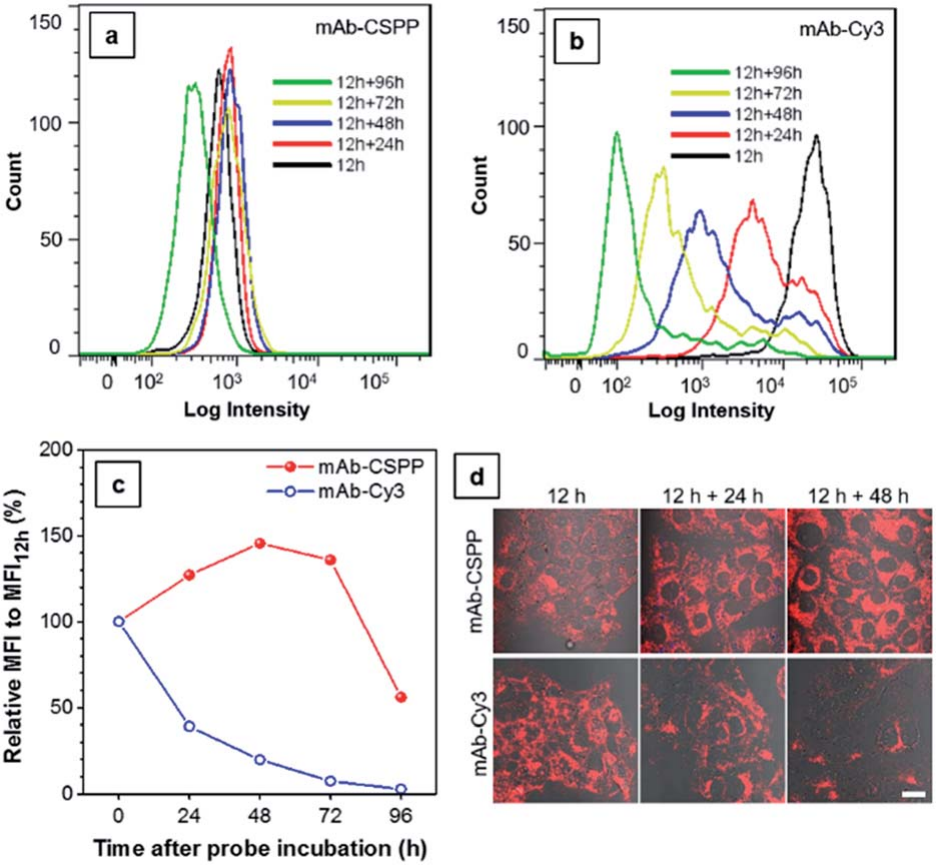
Flow cytometric analysis of HCC827 cancer cells incubated with 10 μg mL^–1^ of (a) mAb–CSPP and (b) mAb–Cy3 at different time intervals. (c) Plot of relative fluorescence intensity (MFI/MFI_12 h_), where MFI_12 h_ is the mean fluorescence intensity at 12 h incubation. Conditions: for mAb–Cy3, *λ*_ex_ = 561 nm, detection with bandpass filter of 583 ± 7.5 nm. For CSPP, *λ*_ex_ = 405 nm, detection with bandpass filter of 610 ± 10 nm. (d) The merged confocal images of the bright field and fluorescence images of cells incubated with mAb–dye conjugates for 12 h, and cells further incubated in fresh medium for 24 and 48 h. Conditions: for mAb–Cy3, *λ*_ex_ = 560 nm, emission filter = 563–700 nm. For mAb–CSPP, *λ*_ex_ = 405 nm, emission filter = 550–700 nm. Scale bar: 20 μm.

The biostability of a fluorescent probe is a pivotal requirement for *in vitro* and *in vivo* long-term imaging applications.^59^ Once the fluorophore is conjugated to the antibody, the fluorescence may be compromised by catabolism. In order to assess the biostability of the mAb–dye conjugates, HCC827 cells were first incubated with the antibody conjugates for 12 h and then with a fresh culture medium for the required time. Afterwards, they were subjected to analysis using a flow cytometer (Fig. 8a and b) and LSCM (Fig. 8d) every 24 h. The results from flow cytometry showed that the MFI of cells incubated with mAb–Cy3 rapidly decreased with time (Fig. 8c). Compared with the MFI at 12 h, the relative values for cells cultured for further 24, 48, 72 and 96 h were 39%, 20%, 7.4%, 2.7%, respectively. On the contrary, the MFI of cells incubated with mAb–CSPP increased to its maximum value at 48 h with a relative MFI of 146% (Fig. 8c). As EGFR downregulation activated by cetuximab is very slow and only apparent after 24 h (60% EGFR remain on the cell membrane).^45^ Therefore, when prolonging the time course from 24 h to 96 h, more activated EGFR would thus be internalized and degraded. It was plausible that the fluorescence increase for cells incubated with mAb–CSPP could be attributed to the receptor-mediated endocytosis into the lysosome and the accumulation of CSPP catabolites in mitochondria after the probe degradation. The fluorescence decreased at 72 h and continued down to a relative MFI of 56% at 96 h, probably due to the diminishment of dye molecules in each cell by cell proliferation. Additionally, the fluorescence enhancement was also revealed in the LSCM images of cells incubated with mAb–CSPP on both day 1 (12 h + 24 h) and day 2 (12 h + 48 h) (Fig. 8d). On the contrary, the fluorescence of cells incubated with mAb–Cy3 reduced largely (Fig. 8d) due to the fast diffusion from the cells after the probe degradation. Thus, mAb–CSPP not only possesses the capability of long-term cell retention, attributed to the accumulation of CSPP residues in mitochondria after catabolism, but also its fluorescence can be further enhanced due to the continuous endocytosis and strong restriction of intramolecular rotation of CSPP in mitochondria. In contrast, mAb–Cy3 showed very short cell retention characteristics due to the rapid cell leakage.

## Conclusions

In summary, we designed a new water-soluble and red-emissive AIEgen, with low cytotoxicity, to be utilized for antibody labelling. The resulting antibody-conjugated AIE probe was successfully applied in “wash-free” and “turn-on” imaging. The probe demonstrated the advantages of a low background signal, long-term cell retention, a large Stokes shift, and good photostability. The probe showed almost no emission in normal cells and in probe medium but was highly emissive in specific cancer cells. Such a distinct difference in emission may enable the probe to differentiate tumors from normal tissues when used in endoscopy and surgical guidance. The characteristics of long-term biostability and good photostability of the probe allowed for long-term cell tracking that could not be visualized before. Due to the rapid advancement of cancer immunotherapy, monoclonal antibodies have been found very useful in treating cancers. It is in demand to monitor the spatiotemporal distribution and the working mechanism of antibody targets in real-time or *in vivo*, thus the mAb–AIEgen conjugates would be a good choice. In future work, water-soluble AIEgens with the property of generating reactive oxygen species or with near infrared emission will be conjugated with proteins capable of activating receptor-mediated endocytosis in cancer cells, such as antibodies specific to estrogen receptors, progesterone receptors and human epidermal growth factor receptor 2. Therefore, the new strategy of preparing “turn-on” protein–AIEgen probes will have a potentially broad range of applications, such as cancer diagnosis with a high TBR, cancer therapy, tracking cell dynamics in real-time, *etc*. This work will inspire more marvellous research in the fields of AIE and bioimaging.

## Experimental procedures

### Materials and instruments

The monoclonal antibody, cetuximab, was isolated from a market solution (Erbitux solution for injection 5 mg mL^–1^, Merck KGaA) *via* ultrafiltration. Sulfo-cyanine 3-NHS ester was from Lumiprobe Corporation (Florida, USA). Phosphate buffered saline solution (PBS, pH 7.4, containing 0.138 M NaCl, 2.7 mM KCl, 10 mM Na_2_HPO_4_, 2 mM KH_2_PO_4_) was obtained from Sigma. Centrifugation was performed using a refrigerated centrifuge (CT15RE tabletop centrifuges, Hitachi Koki Co., Ltd.). The UV/vis spectra were recorded on a spectrophotometer (Biochrom 4060). The fluorescence measurements were carried out on a Perkin-Elmer (LS-55) fluorescence spectrometer. The average particle size and size distribution of the samples were determined using a Brookhaven ZetaPlus potential analyzer (Brookhaven instruments corporation, USA). For the synthetic protocols of CSPP and CSPP-NHS ester, see the ESI.†

### Bioconjugation

The CSPP-NHS ester was dissolved in DMSO to obtain a 10 mM stock solution. Cetuximab (1 mg, 6.8 nmol, 450 μL) was mixed with 50 μL of 1 M Na_2_HCO_3_ stock solution for 5 min. Then CSPP-NHS ester (54.4 nmol) was added. After incubation at 4 °C overnight, the mixture was firstly dialyzed with a dialysis tube (Pur-A-Lyzer, 12–14 kDa MWCO, capacity of 0.1–3 mL, Sigma) in PBS at 4 °C for 5 h, and then was concentrated ultrafast at 14 000 rpm for 15 min in an ultrafiltration tube (Amicon ultra-0.5 centrifugal filter devices, nominal molecular weight limit of 50 kDa) three times and purified with a Sephadex column (illustra NAP-5 columns, Sephadex G-25 DNA, GE Healthcare) using PBS as eluent. The obtained solution was concentrated ultrafast to get the stock solution of the conjugates. The absence of unbound dye in the conjugate solutions was confirmed by reversed-phase thin layer chromatography (RP-C18 TLC). The protein concentration was measured through absorption using the UV-vis system to confirm the numbers of fluorophore molecules conjugated to each cetuximab molecule. The dye–protein (D/P) ratio was determined using the method as described before.^37^,^60^ The molar absorptivity of cetuximab (174 000 L mol^–1^ cm^–1^) was taken from ref. 61. Sulfo-cyanine 3-NHS ester conjugated antibody was synthesized in a similar manner to that described above. The number of dye molecules per antibody was about 3.

### SDS-PAGE of antibody conjugates

As a quality control of the bioconjugation, sodium dodecyl sulfate-polyacrylamide gel electrophoresis (SDS-PAGE) of conjugates was performed as previously described.^62^ SDS-PAGE was carried out using a 4% stacking and a 10% separating gel in a Mini-Protein II apparatus (Bio-Rad Laboratories, Inc.). Samples were prepared in Tris buffer containing SDS, bromophenol blue, glycerol, and 2% β-mercaptoethanol to reduce the disulfide bond in antibody. Amersham ECL Plex fluorescent rainbow marker was used as a standard protein marker. After electrophoresis at 40 V for about 45 min followed by 80 V for about 2 h, the gel was imaged with a Typhoon TRIO System (GE Medical System) using a fluorescence channel of 532 nm (Cy3 channel) and 635 nm (Cy5 channel), and also visualized with a ChemiDoc Imaging System (Bio-Rad Laboratories, Inc.) using the UV channel. The images were merged using Image-Pro Plus 6.0 software (public software from Media Cybernetics, ; http://www.mediacy.com/). Then, the gel was stained with Coomassie Brilliant Blue, and pictures were taken using a digital camera.

### Cell culture

All cells were purchased from ATCC. HCC827 (human non-small cell lung cancer with EGFR overexpression) and NCI–H23 cells (human non-small cell lung cancer with low EGFR expression) and cultured in RPMI-1640 (Life Technologies, Gaithersburg, MD) with 1% penicillin–streptomycin and 10% heat-inactivated FBS at 37 °C in a humidified incubator containing 5% CO_2_. Normal cells (HEK-293, COS-7, NIH 3T3 and MDCK.2) were grown in DMEM (Life Technologies, Gaithersburg, MD) containing 1% penicillin–streptomycin and 10% heat-inactivated FBS. HeLa cells were cultured in MEM (Life Technologies, Gaithersburg, MD) containing 1% penicillin–streptomycin and 10% heat-inactivated FBS. The culture medium was changed every other day and the cells were collected by treating with 0.25% (w/v) trypsin–0.53 mM EDTA solution after they reached confluence.

### Cytotoxicity study

Cell Counting Kit-8 (CCK-8) based on utilizing WST-8 [2-(2-methoxy-4-nitrophenyl)-3-(4-nitrophenyl)-5-(2,4-disulfophenyl)-2*H*-tetrazolium, monosodium salt] was used to evaluate the cytotoxicity of CSPP dye. HCC827 and NCI-H23 cells were seeded in a 96-well plate at a density of 5000 cells per well. After 16 h incubation, the cells were exposed to a series of doses of CSPP (0–50 μM) in culture medium at 37 °C for 24 h. Next, the dye solution in wells were removed and 100 μL of culture medium and 10 μL of CCK-8 solution were added into each well. After incubation for 3 h at 37 °C, the absorbance at 450 nm was recorded using a Perkin-Elmer Victor plate reader. Each group had six parallel samples.

### Cell imaging

Cells (1 × 10^5^ cells per mL) were seeded on a 35 mm Petri dish with a cover slip or a plasma-treated 25 mm round cover slip mounted to the bottom of a 35 mm Petri dish with an observation window. After cell culture for two days, antibody–dye conjugates (10 μg mL^–1^) were added into cells and they were incubated for the required time. Then the cells were imaged directly or after incubation in a new culture medium for a certain time. For wash-free imaging using fluorescence microscopy, FluoroBrite DMEM (Life Technologies, Gaithersburg, MD) featuring a background fluorescence that is comparable to PBS was used to prepare the probe medium to avoid the background fluorescence from the culture medium. Fluorescence pictures were taken using an Olympus BX41 microscope (Olympus America, Inc., Melville, NY). Different excitation and emission filters were used for each dye: for Cy3, *λ*_ex_ = 510–550 nm, dichroic mirror = 570 nm, long-pass emission filter; for CSPP, *λ*_ex_ = 400–440 nm, dichroic mirror = 455 nm, long-pass emission filter. Exposure time is 1 s for all pictures.

To track the endocytosis process of the mAb–dye conjugates, cells were incubated in probe medium (10 μg mL^–1^ of mAb–dye conjugates in FluoroBrite DMEM) for different times. Cells incubated with mAb–CSPP were imaged in probe medium without washing, while cells incubated with mAb–Cy3 were imaged in FluoroBrite DMEM after washing. These images were acquired using a LSCM (Zeiss Laser Scanning Confocal Microscope; LSM7 DUO) equipped with the accessory of a Bioptechs Focht Chamber System 2 (FCS 2) and analyzed using ZEN 2009 software (Carl Zeiss). To investigate the intracellular localization of mAb–CSPP, the co-localization studies were performed using a lysosomal marker (LysoTracker Green DND-26, Invitrogen Co., Carlsbad, CA) and a mitochondrion marker (MitoTracker Orange, Invitrogen Co., Carlsbad, CA). MitoTracker was added 15 min prior to imaging, and LysoTracker was added 5 min ahead of imaging. Different excitation and emission filters were used for each dye: for CSPP, *λ*_ex_ = 405 nm, emission filter = 550–700 nm; for Cy3, *λ*_ex_ = 560 nm, emission filter = 563–700 nm, for LysoTracker Green, *λ*_ex_ = 488 nm, emission filter = 495–535 nm, for MitoTracker Orange, *λ*_ex_ = 560 nm, emission filter = 565–585 nm.

For the photostability test, the cells were imaged using a LSCM. mAb–CSPP was excited at 405 nm (10% laser power) and the fluorescence was collected between 550 and 700 nm. As a comparison, the excitation power from 405 nm for mAb–CSPP and from 560 nm for mAb–Cy3 was unified as 32 μW. The fluorescence of mAb–Cy3 was collected between 563 and 700 nm.

To prove that the mAb–CSPP probe was not emissive on the cell membrane, HCC827 cells were incubated with mAb–CSPP or mAb–Cy3 conjugates for 25 min at 37 °C. After cell fixation, permeabilization, and blocking, the cells were stained with goat F(ab′)2 anti-human IgG F(ab′)2 (FITC) (Abcam Inc., Cambridge, MA, USA). Pictures were taken in PBS immediately after immunofluorescence staining, or taken after cells were mounted in Fluoromount Aqueous Mounting Medium (Sigma-Aldrich, St. Louis, MO, USA) followed by immunofluorescence staining. FITC was excited at 488 nm and the fluorescence was collected between 495 nm and 535 nm. The excitation and emission filters were the same as described above for CSPP and Cy3. The laser intensity, pinhole, gain, and offset settings for the images of CSPP before and after mounting medium disposal were kept the same.

### The analysis by flow cytometer

Flow cytometry was performed in order to investigate the endocytosis-induced fluorescence enhancement and the *in vitro* biostability of the mAb–CSPP and mAb–Cy3 conjugates. HCC827 cells were plated on a 35 mm Petri dish and cultured for two days. Then, cells were added to mAb–CSPP or mAb–Cy3 conjugates (10 μg mL^–1^) to incubate for 1 h or 12 h. Next, the cells were washed two times and cultured in a new RPMI 1640 medium for different times (11 h, 24 h, 48 h, 72 h or 96 h). The cells were trypsinized to measure the *in vitro* fluorescence intensity of cells using a flow cytometer (FACS Calibur, BD BioSciences, San Jose, CA, USA). All data were analyzed with CellQuest software (BD BioSciences). The mAb–CSPP was excited at 405 nm and its fluorescence signal was collected using a bandpass filter of 610 ± 10 nm. mAb–Cy3 was excited at 561 nm and detected with a bandpass filter of 583 ± 7.5 nm. The mean fluorescence intensity (MFI) was used to show the endocytosis-induced fluorescence enhancement and the *in vitro* biostability of the mAb–dye conjugates.

### Antibody digestion and cell lysis

The antibody denaturation and digestion was conducted according to a reported method with a little change.^56^ Specifically, the mAb–CSPP conjugates of 0.4 mg mL^–1^ were pre-treated with 4 M of urea and 1.6 mg mL^–1^ Proteinase K at 37 °C for 12 h.

Cell lysate was prepared by ultrasonicating HeLa cells of 8 × 10^5^ per mL in water for 30 min.

## Live subject statement

All experiments were performed in compliance with the relevant laws and institutional guidelines. And the institutional committees have approved the experiments. Informed consent was obtained for any experimentation with human subjects.

## Conflicts of interest

There are no conflicts of interest to declare.

## Supplementary Material

Supplementary information

Crystal structure data
