# Supplementary material for: A red-emissive antibody–AIEgen conjugate for turn-on and wash-free imaging of specific cancer cells
Source: Chem Sci. 2017 Aug 17;8(10):7014–24. doi: 10.1039/c7sc01054k (PMC6103257; doi:10.1039/c7sc01054k)
Supplement: Supplementary file 1 [file SC-008-C7SC01054K-s001.pdf]

Electronic Supplementary Material (ESI) for Chemical Science.
This journal is © The Royal Society of Chemistry 2017

Electronic Supplementary Information

Red-emissive antibody-AIEgen conjugate for turn-on and wash-free imaging of specific cancer cell†

Xiujuan Shi,‡[ab] Chris Y. Y. Yu,‡[ab] Huifang Su,[ab] Ryan T. K. Kwok,[ab] Meijuan Jiang,[ab] Zikai He,[ab] Jacky W. Y. Lam[ab] and Ben Zhong Tang*[abc]

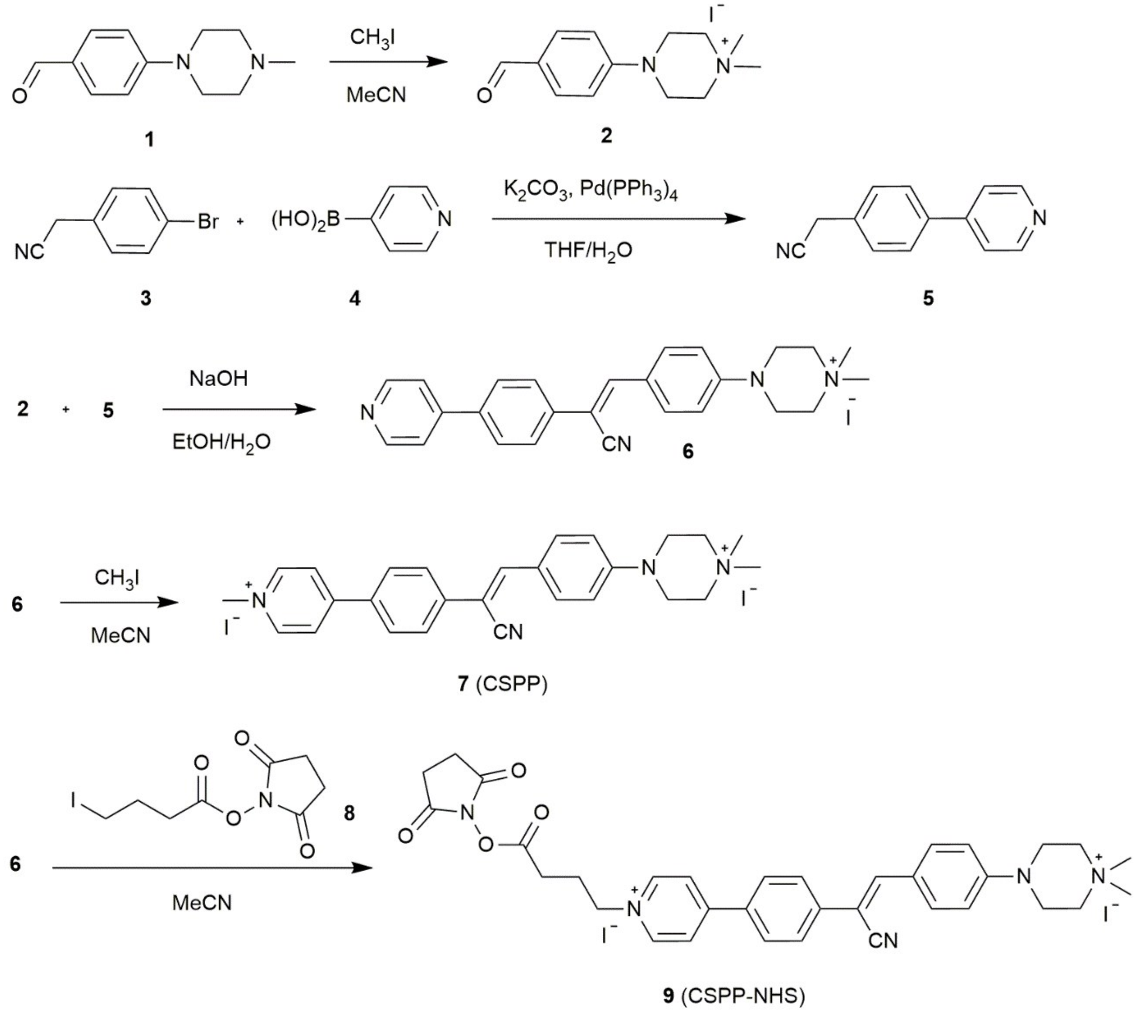

Fig. S1 Synthetic route for CSPP (**7**) and CSPP-NHS (**9**).

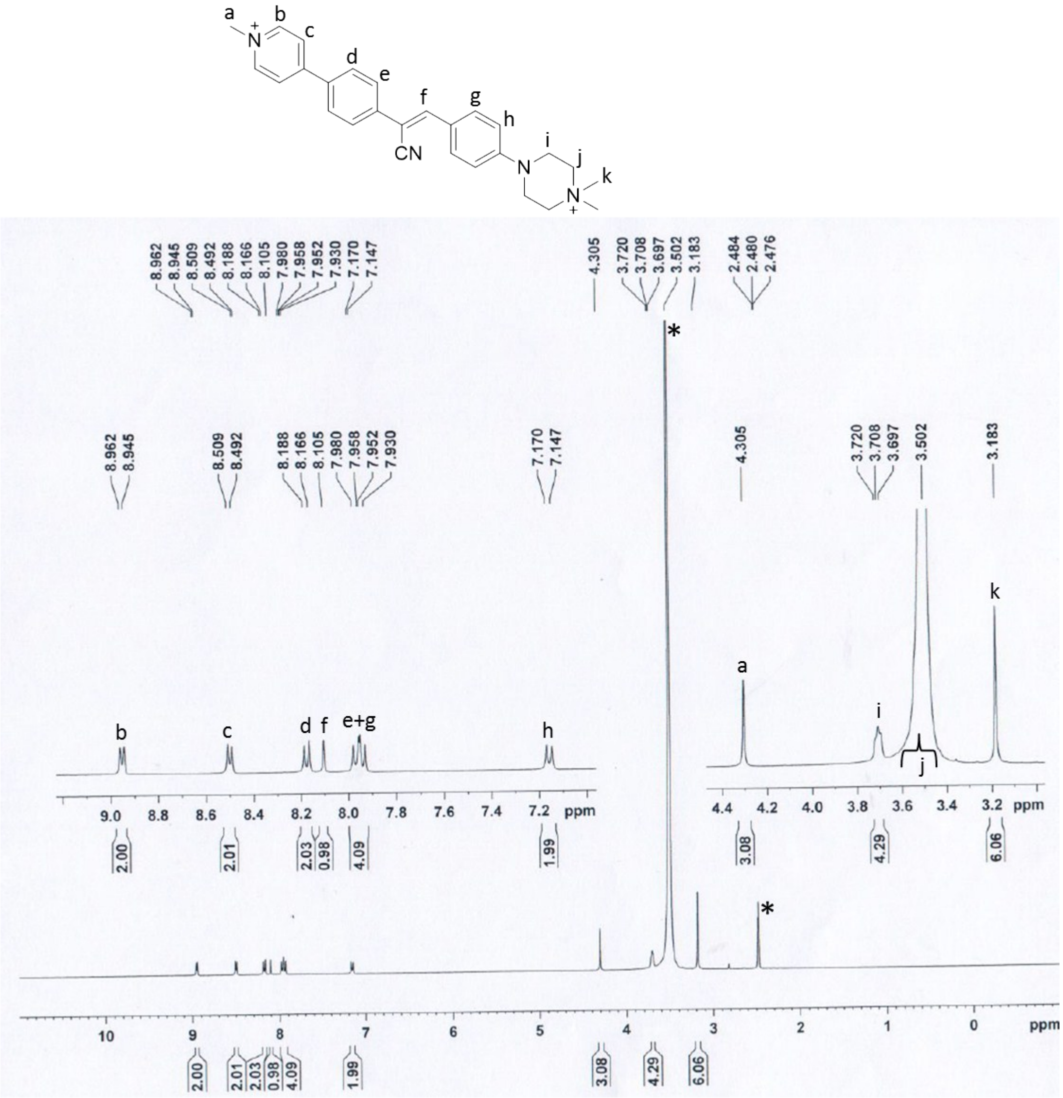

Fig. S2 ^{1}H NMR spectrum of CSPP in DMSO-d_6.

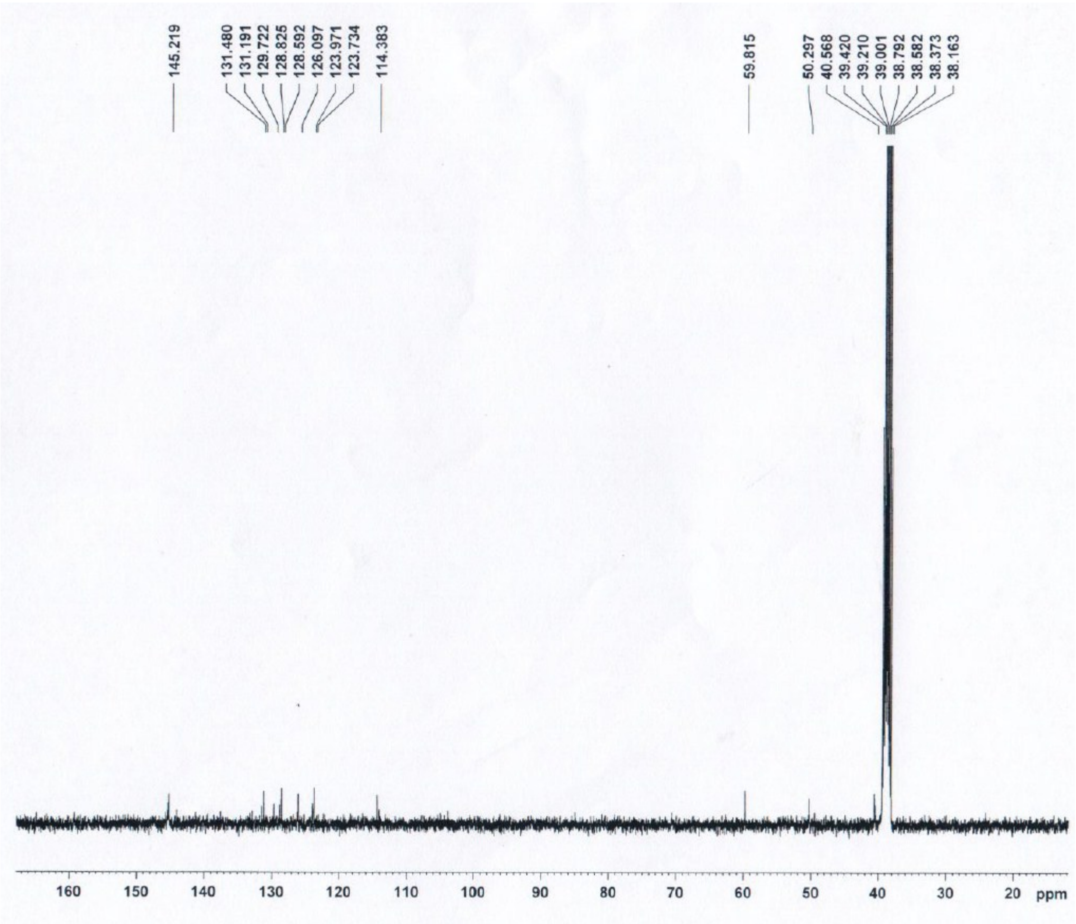

Fig. S3 ^{13}C NMR spectrum of CSPP in DMSO-d_6.

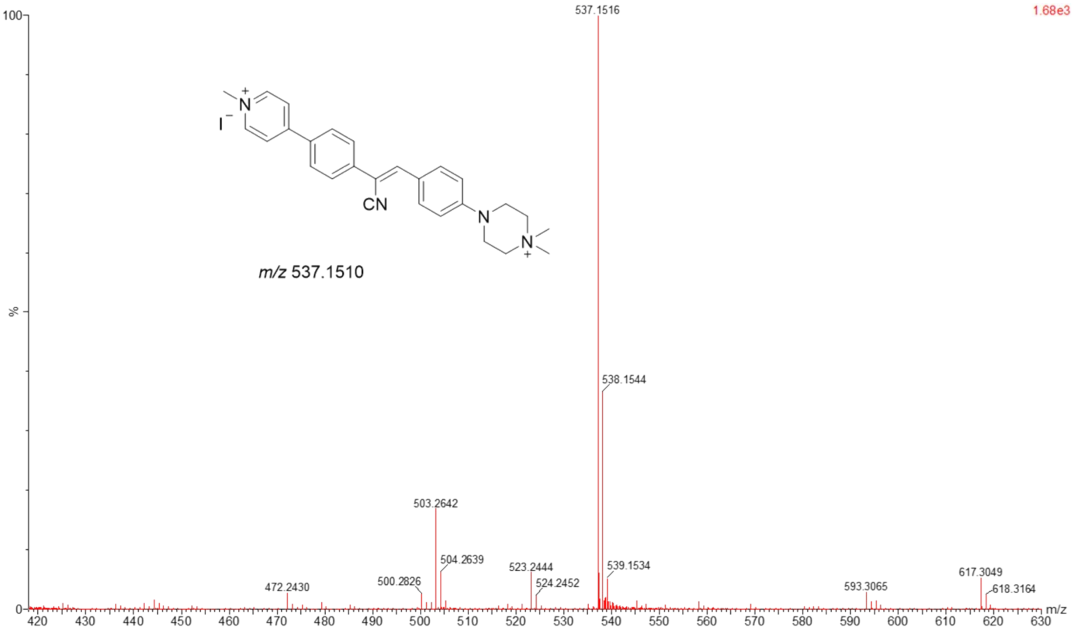

Fig. S4 High resolution mass spectrum of CSPP.

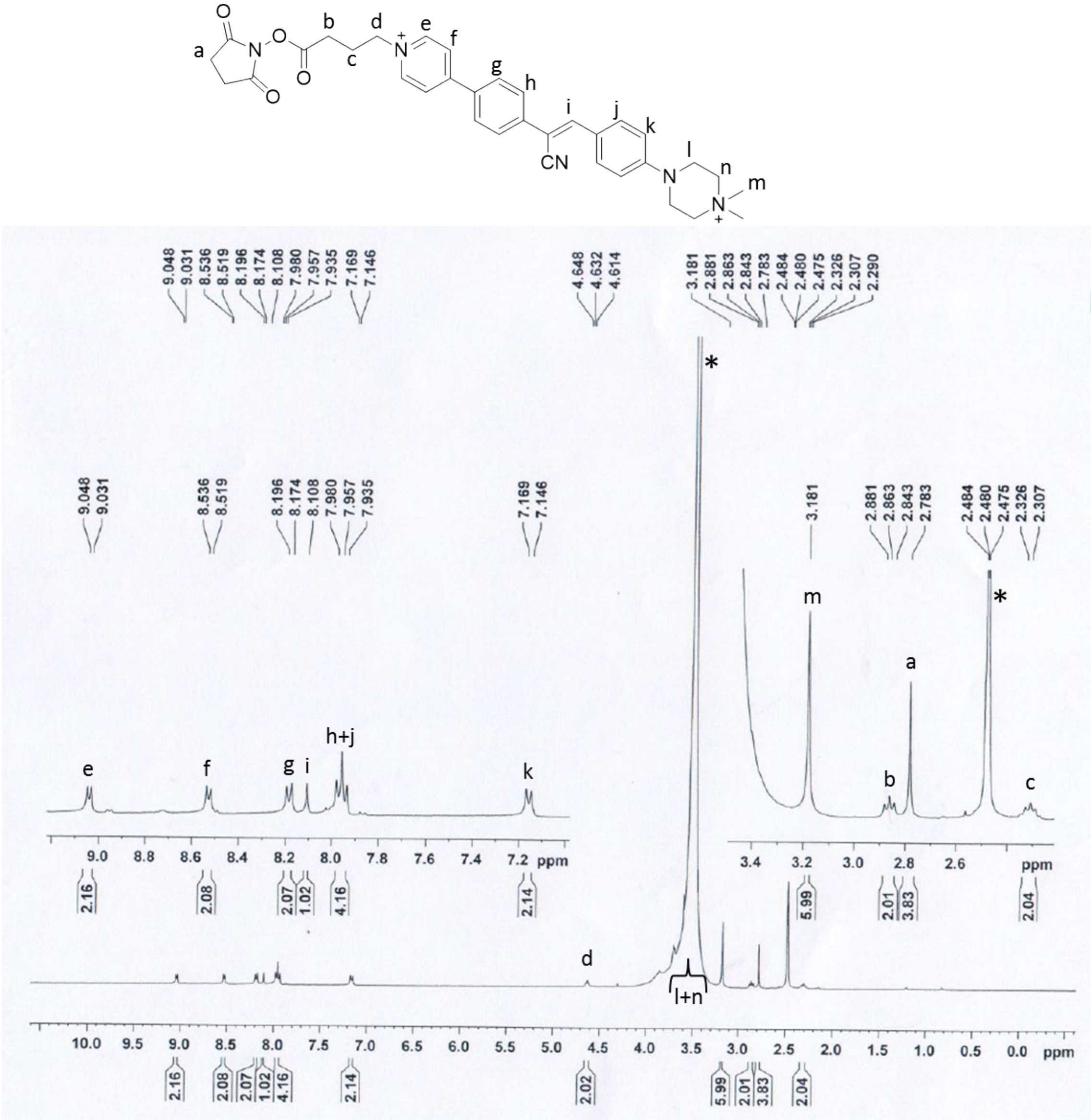

Fig. S5 ^{1}H NMR spectrum of CSPP-NHS in DMSO-d_6.

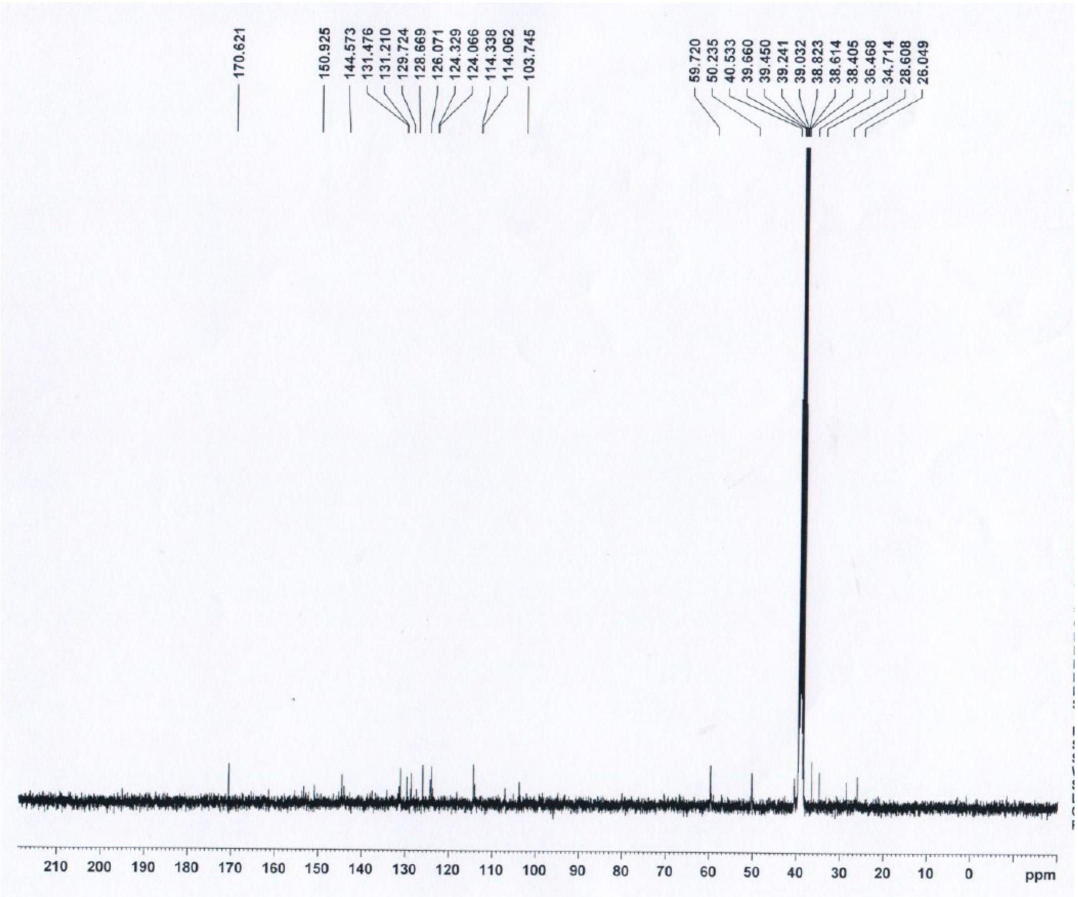

Fig. S6 ^{13}C NMR spectrum of CSPP-NHS in DMSO-d_6.

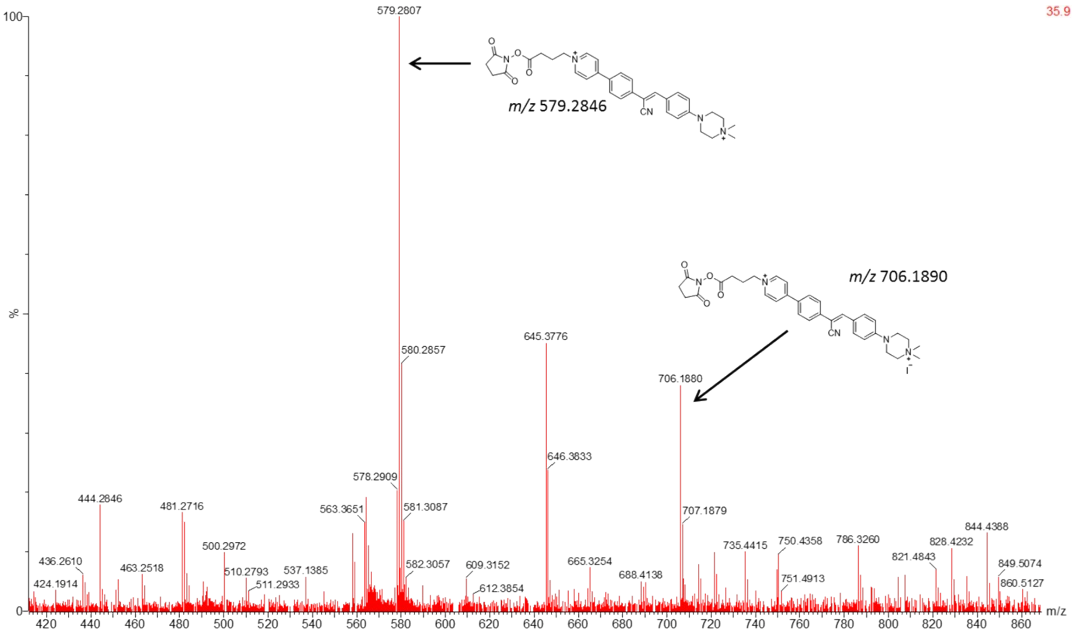

Fig. S7 High resolution mass spectrum of CSPP-NHS.

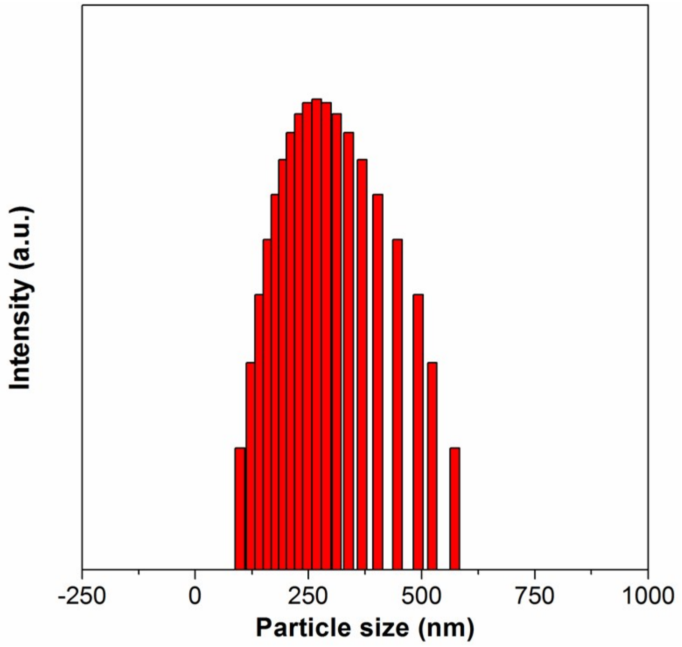

Fig. S8 Hydrodynamic particle size of CSPP aggregates formed in the water/iPrOH mixture with 99% iPrOH fraction measured by the method of dynamic light scattering.

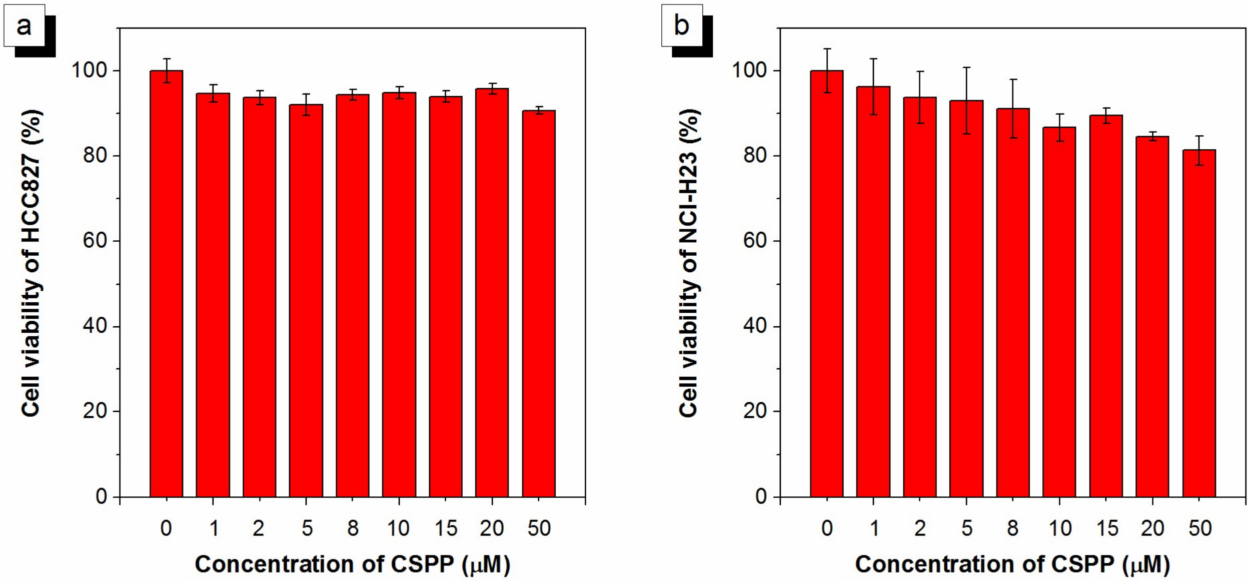

Fig. S9 Cell viability of HCC827 and NCI-H23 cells incubated in culture medium with different concentrations of CSPP for 24 h. Data given are the mean ± SD (*n* = 4).

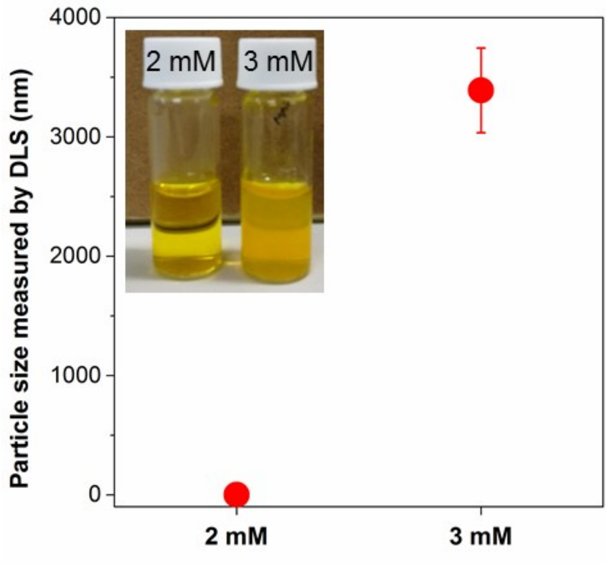

Fig. S10 The particle size of CSPP in water with a concentration of 2 mM and 3 mM measured by DLS. Data were the mean ± SD (n = 5). The inserted pictures showed whether the solution of CSPP was turbid or not. The black line from behind indicates that the solution of 2 mM was clear, but the solution of 3 mM was turbid.

Table S1 Parameters of mAb-CSPP and mAb-Cy3 conjugates[a]

	D/P	CF@ 280 nm	ε (L mol^{-1} cm^{-1})	λ_{abs} (nm)	λ_{em} (nm)	Stokes shift (nm)
mAb-CSPP	3.0	0.3	26800	405	624	219
mAb-Cy3	3.5	0.06	162000	550	567	17

[a]Abbreviation: D/P = dye/protein molar ratio, CF = correction factor for the fluorophore's contribution to the absorbance at 280 nm, ε = molar absorptivity, λ_{abs} = maximum absorption wavelength, λ_{em} = maximum emission wavelength.

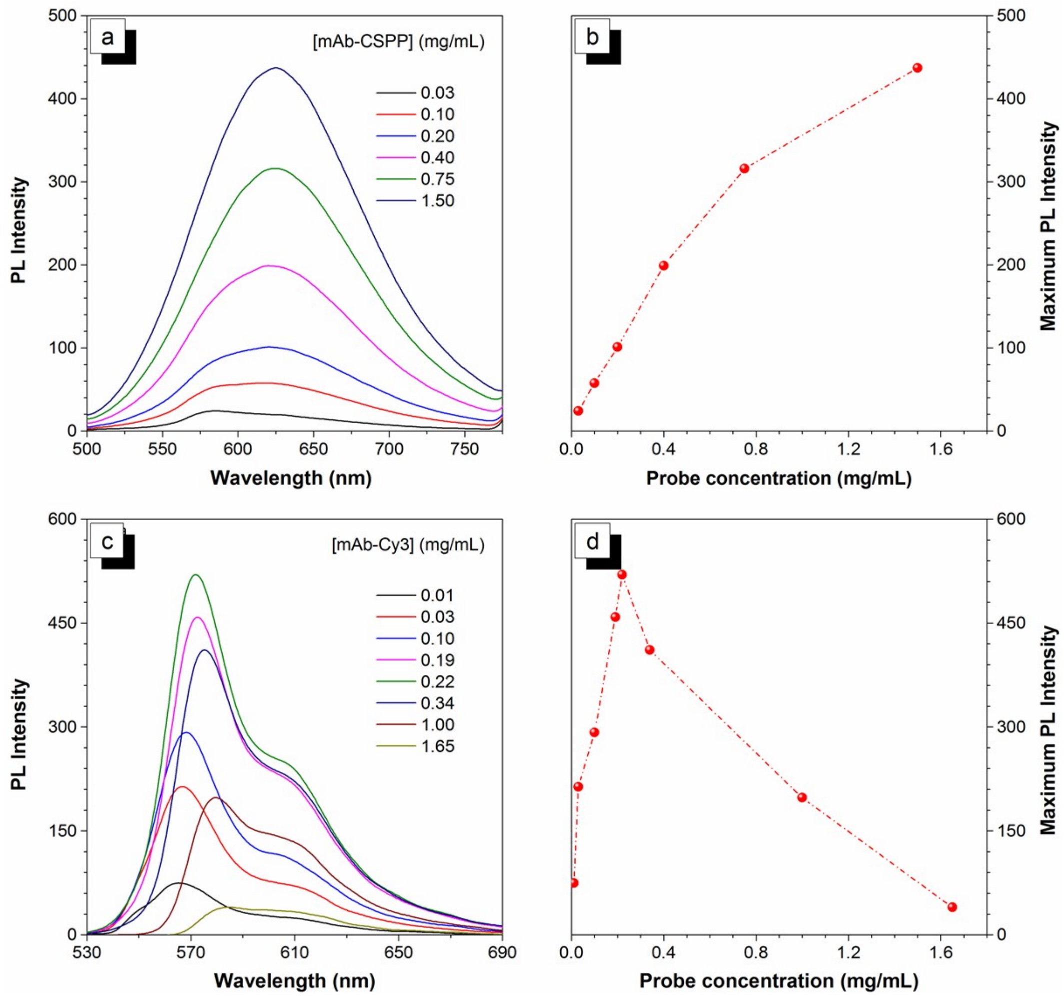

Fig. S11 (a and c) PL spectra and (b and d) plots of maximum fluorescence intensity of (a and b) mAb-CSPP and (c and d) mAb-Cy3 conjugates in PBS versus the probe concentration.

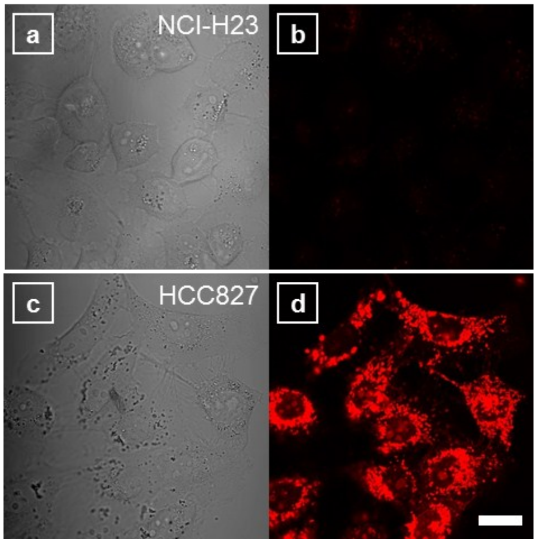

Fig. S12 (a and c) Bright-field and (b and d) confocal fluorescence images of (a and b) EGFR-negative NCI-H23 cells and (c and d) EGFR-positive HCC827 cells incubated with 10 µg/mL of mAb-CSPP conjugate for 24 h. λ_{ex}: 405 nm; emission filter: 550–700 nm; scale bar: 20 µm.

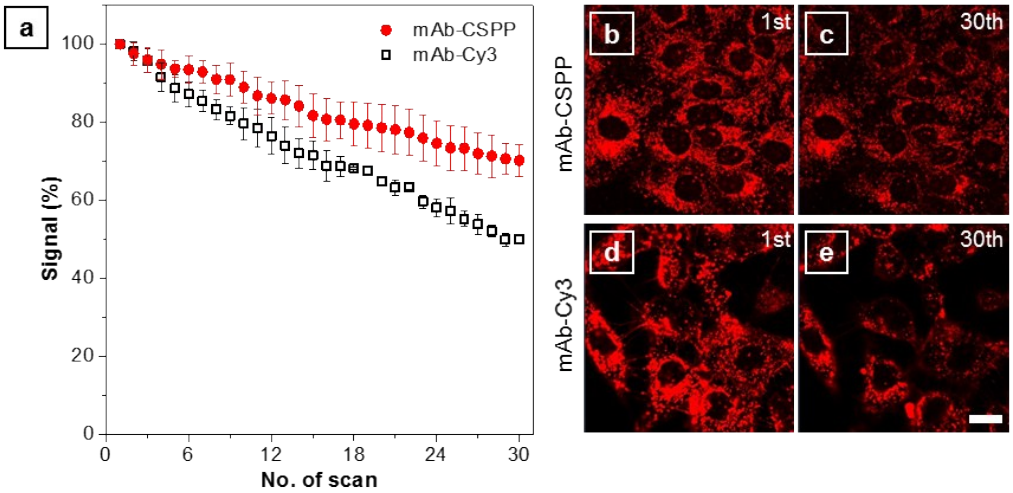

Fig. S13 (a) Loss in fluorescence signal of (red circle) mAb-CSPP- and (blank square) mAb-Cy3-stained HCC827 cells after continuous scanning using LSCM. Data are the mean ± SD (n = 4). (b–e) LSCM images of HCC827 cells stained with (b and c) mAb-CSPP and (d and e) mAb-Cy3 conjugates at the 1st and 30th scans. Concentration: 10 µg/mL; laser power: 32 µW; scale bar: 20 µm. For mAb-CSPP: λ_{ex} = 405 nm; emission filter = 550–700 nm. For mAb-Cy3: λ_{ex} = 560 nm, emission filter = 563–700 nm.

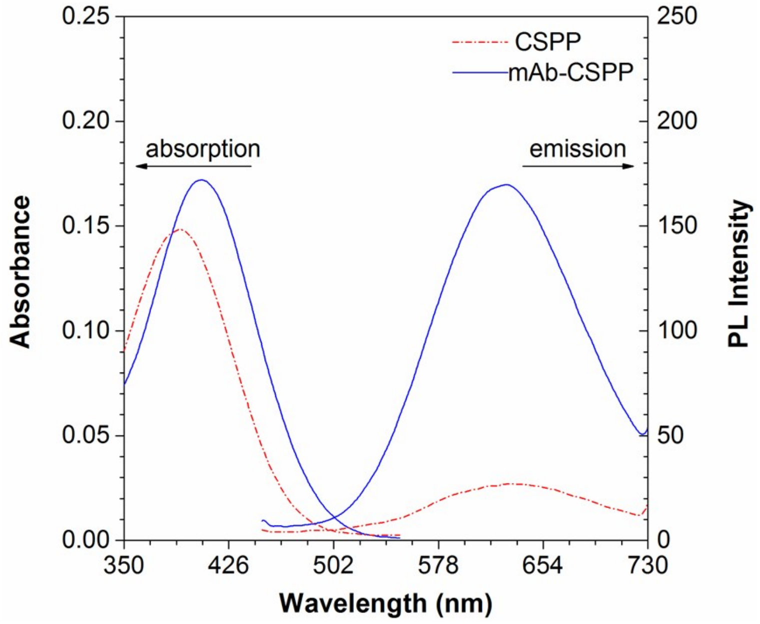

Fig. S14 Comparison of the absorption and emission of CSPP molecule (5.5 μM) and mAb-CSPP conjugate (0.38 mg/mL mAb containing ~6.4 μM CSPP molecule) in PBS buffer.

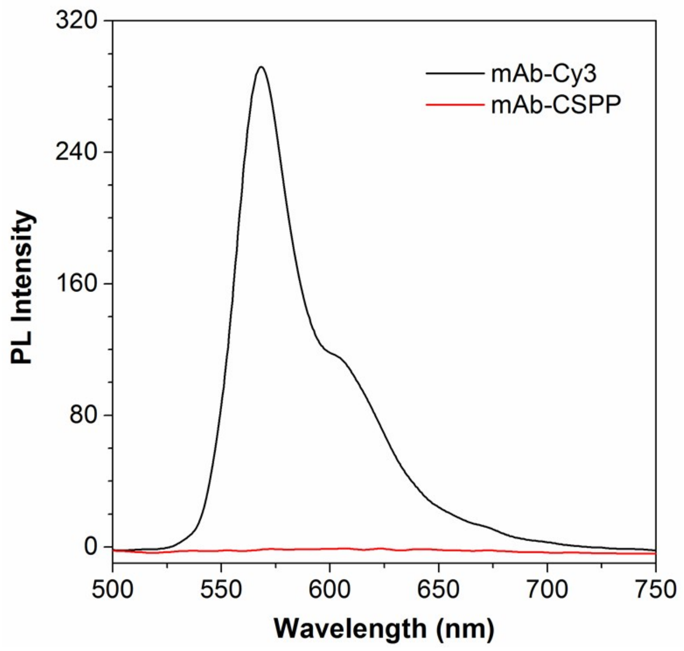

Fig. S15 PL spectra of mAb-Cy3 and mAb-CSPP conjugates in PBS buffer measured at same conditions. Concentration: 0.1 mg/mL.

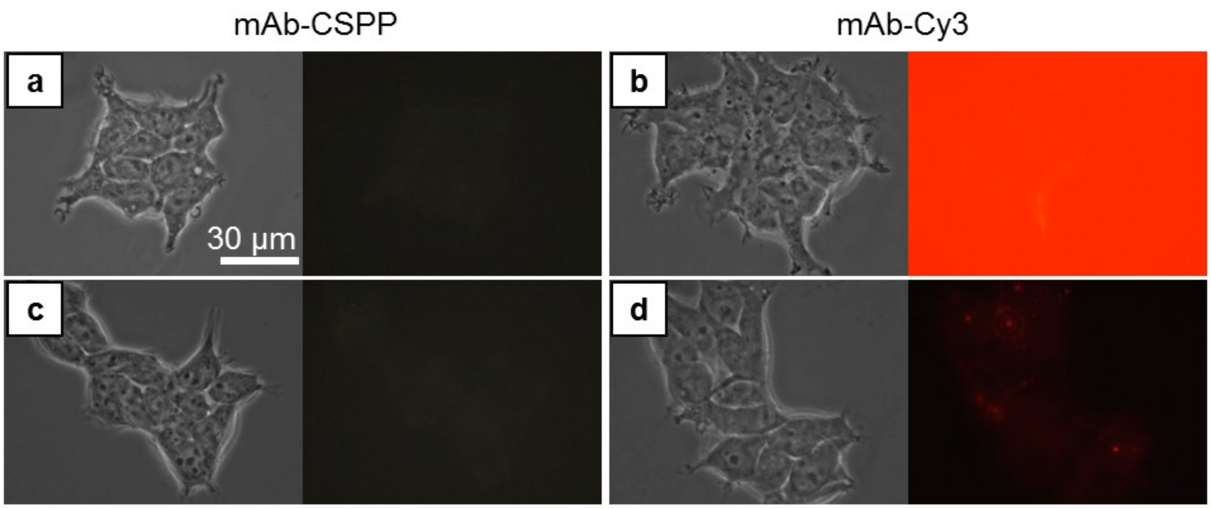

Fig. S16 Bright-field and fluorescent images of HEK-293 normal cells incubated with 10 µg/mL of mAb-CSPP and mAb-Cy3 conjugates for 12 h. Images were taken (a, b) in probe medium without washing and (c, d) in PBS after washing. Conditions: for mAb-Cy3, λ_{ex} = 510–550 nm, dichroic mirror = 570 nm; for mAb-CSPP, λ_{ex} = 400–440 nm, dichroic mirror = 455 nm. Exposure time = 1 s; scale bar = 30 µm.

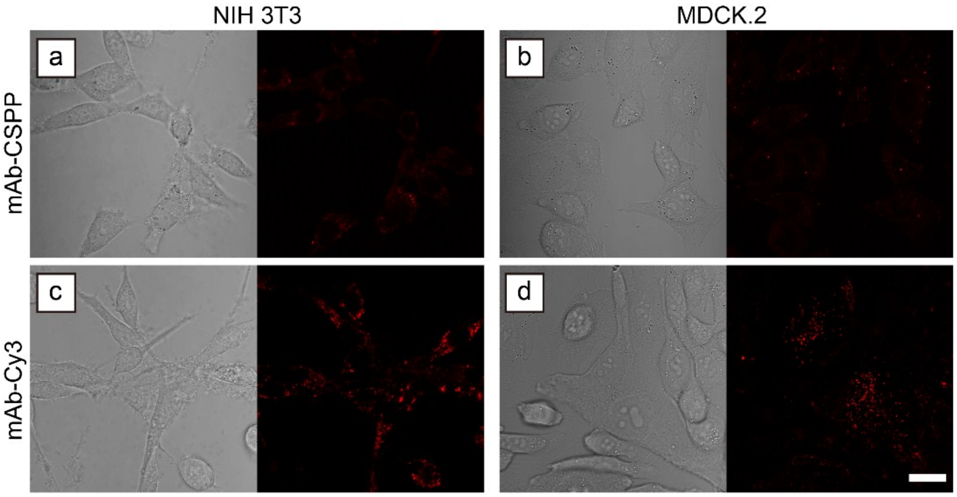

Fig. S17 LSCM images of (a and c) NIH 3T3 and (b and d) MDCK.2 normal cells incubated with 10 µg/mL of (a and b) mAb-CSPP or (c and d) 10 µg/mL of mAb-Cy3 for 12 h. For mAb-CSPP: λ_{ex} = 405 nm; emission filter = 550–700 nm. For mAb-Cy3: λ_{ex} = 560 nm, emission filter = 563–700 nm. Scale bar: 20 µm.

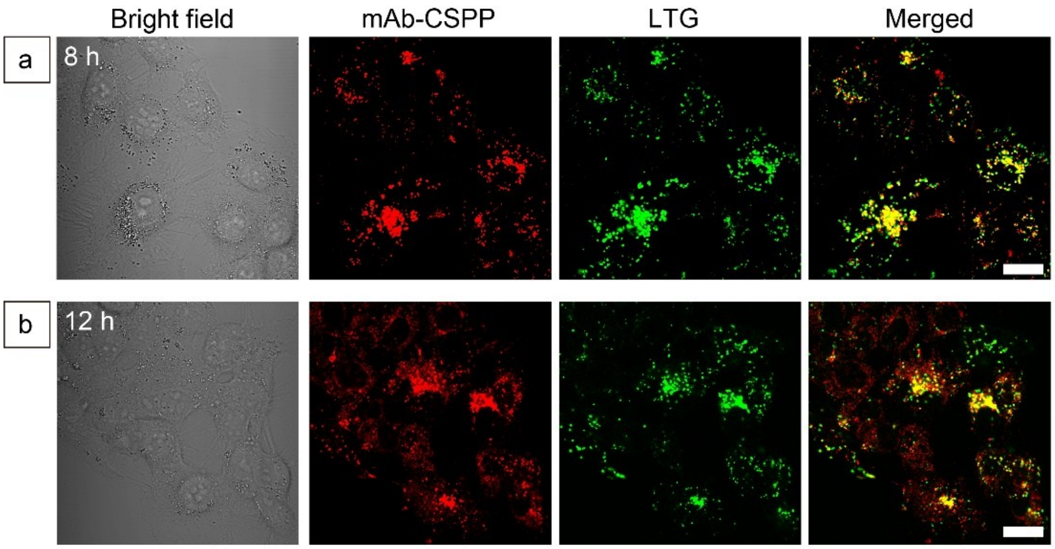

Fig. S18 LSCM images of HCC827 cells incubated with 10 μg/mL of mAb-CSPP conjugate for (a) 8 h and (b) 12 h, followed by staining with LysoTracker Green (LTG) for 5 min. For mAb-CSPP, λ_{ex} = 405 nm, emission filter = 550–700 nm. For LTG, λ_{ex} = 488 nm, emission filter = 495–535 nm. Scale bar: 20 μm.

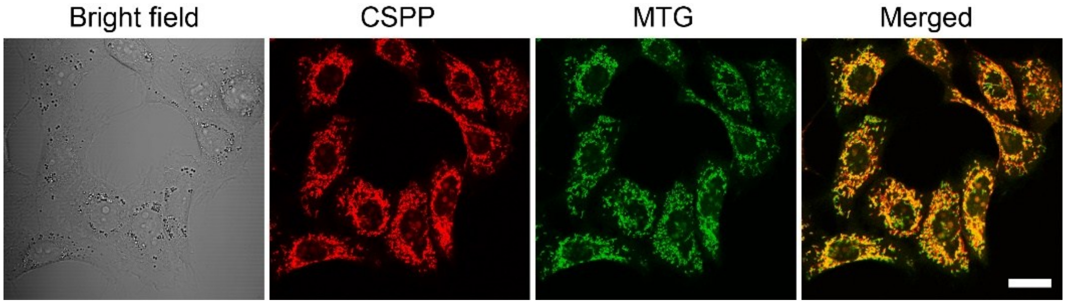

Fig. S19 LSCM images of HCC827 cells stained with 5 μM of CSPP for 8 h followed by staining with MitoTracker Green (MTG) for 15 min. For CSPP, λ_{ex} = 405 nm; emission filter = 550–700 nm. For MTG, λ_{ex} = 488 nm, emission filter = 495–535 nm. Scale bar: 20 μm.

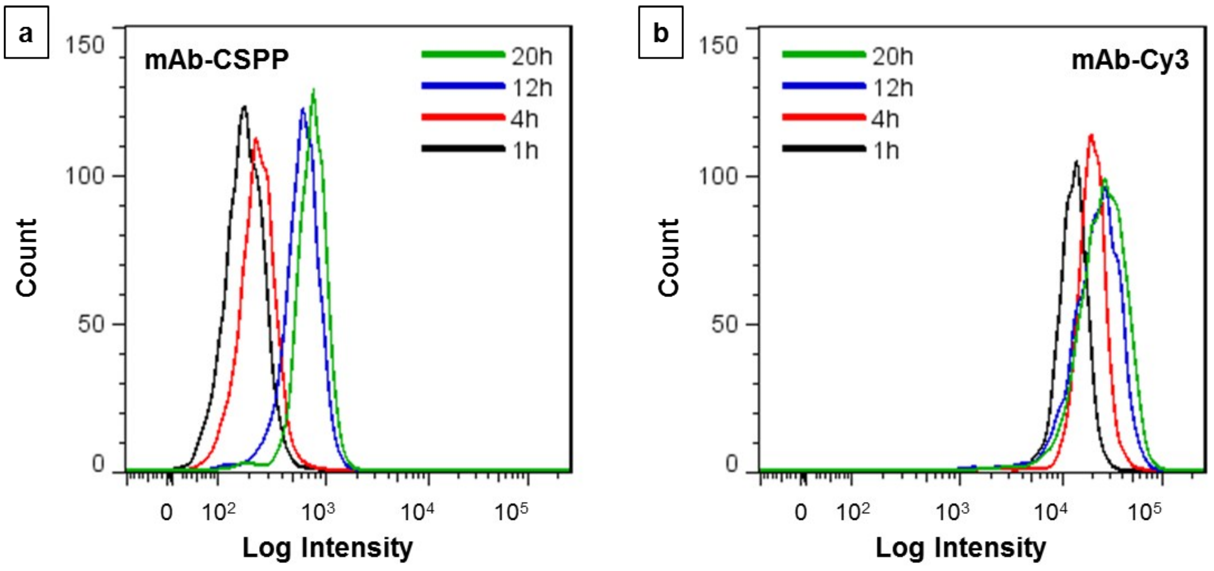

Fig. S20 Flow cytometric analysis of HCC827 cells after incubation with (a) mAb-CSPP and (b) mAb-Cy3 conjugates at different time intervals (1 h, 4 h, 12 h and 20 h). Conditions: for mAb-Cy3, λ_{ex} = 561 nm, detection with bandpass filter = 583±7.5 nm. For mAb-CSPP, λ_{ex} = 405 nm, detection with band-pass filter = 610±10 nm.

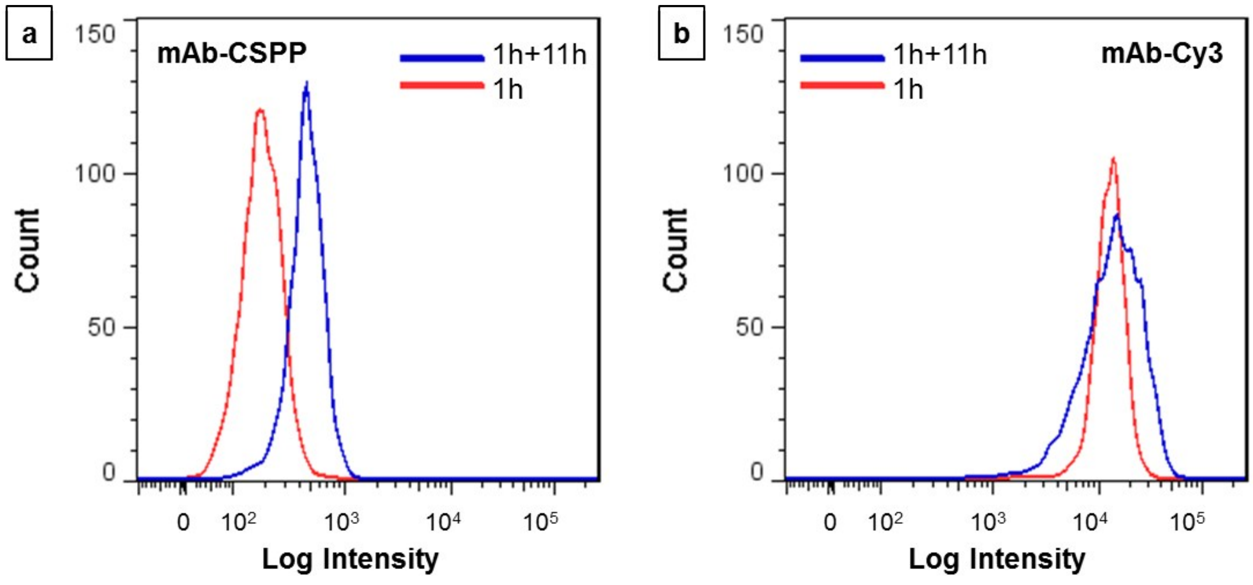

Fig. S21 Flow cytometric analysis of HCC827 cells after incubation with (a) mAb-CSPP and (b) mAb-Cy3 conjugates for 1 h, followed by incubation in fresh medium without antibody probe for 11 h. Conditions: for mAb-Cy3, λ_{ex} = 561 nm, detection with bandpass filter = 583±7.5 nm. For mAb-CSPP, λ_{ex} = 405 nm, detection with band-pass filter = 610±10 nm.

Synthesis of CSPP and CSPP-NHS

4-(4-Methylpiperazin-1-yl)benzaldehyde[1] (**1**) and 2,5-dioxopyrrolidin-1-yl 4-iodobutanoate[2] (**8**) were synthesized according to the previous report. CSPP and CSPP-NHS were prepared according to the synthetic route shown in **Fig. S1** of **ESI**. Detailed procedures are shown below.

Synthesis of Compound **2**: Into a 100 mL two-necked round bottom flask equipped with a condenser was dissolved **1** (0.20 g, 0.98 mmol) in 15 mL of acetonitrile. Iodomethane (0.15 mL) was then added and the mixture was heated to reflux for 4 h. After cooling to room temperature, the mixture was poured into diethyl ether. The pale yellow precipitates formed were filtered by suction filtration to give **2** (0.32 g, 95%). ^{1}H NMR (400 MHz, DMSO-d_6), δ (ppm): 9.76 (s, 1H), 7.78 (d, 2H, J = 8.8 Hz), 7.15 (d, 2H, J = 8.8 Hz), 3.74 (t, 4H, J = 4.4 Hz), 3.54 (t, 4H, J = 4.8 Hz), 3.20 (s, 6H). ^{13}C NMR (100 MHz, DMSO-d_6), δ (ppm): 190.5, 153.3, 131.2, 127.2, 113.8, 59.6, 50.2. HRMS (MALDI-TOF): *m/z* 219.1516 (M^+, calcd. 219.1497).

Synthesis of Compound **5**: Into a 100 mL two-necked round bottom flask equipped with a condenser were added 2-(4-bromophenyl)acetonitrile (**3**, 0.50 g, 2.55 mmol), 4-pyridinylboronic acid (**4**, 0.31 g, 2.55 mmol), potassium carbonate (3.52 g, 25.5 mmol) and $Pd(PPh_3)_4$ (35 mg, 0.03 mmol) in 50 mL of THF and 10 mL of water under nitrogen. The mixture was stirred and heated to reflux overnight. After cooling to room temperature, the mixture was extracted with dichloromethane (DCM) for three times. The organic phase was collected, washed with water and dried over anhydrous sodium sulfate. After solvent evaporation, the crude product was purified by silica-gel column chromatography using DCM/ethyl acetate (v/v = 99:1) as eluent to furnish a white solid as product (0.40 g, 81%). ^{1}H NMR (400 MHz, $CDCl_3$), δ (ppm): 8.69 (d, 2H, J = 6.0 Hz), 7.67 (d, 2H, J = 8.4 Hz), 7.51–7.46 (m, 4H), 3.83 (s, 2H). ^{13}C NMR (100 MHz, $CDCl_3$), δ (ppm): 149.7, 146.7, 137.5, 130.2, 128.1, 127.1, 120.9, 116.9, 22.9. HRMS (MALDI-TOF): *m/z* 194.0914 (M^+, calcd. 194.0844).

Synthesis of Compound **6**: Into a 50 mL round bottom flask were dissolved **5** (0.2 g, 1.03 mmol) and **2** (0.36 g, 1.03 mmol) in 8 mL of ethanol and 2 mL of water. Sodium hydroxide (41 mg, 1.03 mmol) in 2 mL of ethanol was then added slowly into the mixture. After stirring for 2 h, the pale yellow precipitates were filtered, washed with cold ethanol and dried under reduced pressure to give **6** (0.47 g, 87%). ^{1}H NMR (400 MHz, DMSO-d_6), δ (ppm): 8.63 (d, 2H, J = 6.0 Hz), 7.99 (s, 1H), 7.95–7.91(m, 4H), 7.84 (d, 2H, J = 8.4 Hz), 7.76 (d, 2H, J = 6.0 Hz), 7.16 (d, 2H,

J = 8.8 Hz), 3.69 (t, 4H, J = 4.8 Hz), 3.18 (s, 6H). ^{13}C NMR (100 MHz, DMSO-d_6), δ (ppm): 150.1, 144.7, 137.3, 134.4, 131.4, 129.3, 127.6, 127.3, 125.8, 123.6, 121.0, 120.9, 114.1, 107.6, 59.7, 50.1. HRMS (MALDI-TOF): *m/z* 395.2261 (M^+, calcd. 395.2236).

Synthesis of Compound **7** (CSPP): Into a 100 mL two-necked round bottom flask equipped with a condenser was dissolved **6** (50 mg, 0.096 mmol) in 5 mL of acetonitrile. Iodomethane (0.1 mL) was then added and the mixture was heated to reflux for 4 h. After cooling to room temperature, the mixture was poured into diethyl ether. The orange precipitates formed were filtered by suction filtration to give **7** (62 mg, 97%). ^{1}H NMR (400 MHz, DMSO-d_6), δ (ppm): 9.05 (d, 2H, J = 6.8 Hz), 8.54 (d, 2H, J = 6.8 Hz), 8.20 (d, 2H, J = 8.8 Hz), 8.11 (s, 1H), 7.98–7.94 (m, 4H), 7.17 (d, 2H, J = 9.2 Hz), 4.31 (s, 3H), 3.71 (d, 2H, J = 4.8 Hz), 3.18 (s, 6H). ^{13}C NMR (100 MHz, DMSO-d_6), δ (ppm): 145.2, 131.5, 131.2, 129.7, 128.8, 128.6, 126.1, 124.0, 123.7, 114.4, 59.8, 50.3, 40.6. HRMS (MALDI-TOF): *m/z* 537.1516 (M^+, calcd. 537.1515).

Synthesis of Compound **9** (CSPP-NHS): Into a 100 mL two-necked round bottom flask equipped with a condenser, was dissolved **6** (50 mg, 0.096 mmol) in 5 mL of acetonitrile. **8** (36 mg, 0.1152 mmol) was then added and the mixture was heated to reflux until the spot of **6** on TLC plate disappeared. After cooling to room temperature, the mixture was poured into diethyl ether. The orange precipitates formed were filtered by filtration and washed by cold ethanol to give **9** (58 mg, 72%). ^{1}H NMR (400 MHz, DMSO-d_6), δ (ppm): 8.96 (d, 2H, J = 6.8 Hz), 8.51 (d, 2H, J = 6.8 Hz), 8.19 (d, 2H, J = 8.8 Hz), 8.11 (s, 1H), 7.98–7.93 (m, 4H), 7.17 (d, 2H, J = 9.2 Hz), 4.63 (t, 2H, J = 6.4 Hz), 3.18 (s, 6H), 2.86 (t, 2H, J = 7.2 Hz), 2.78 (s, 4H), 2.31 (t, 2H, J = 7.6 Hz). ^{13}C NMR (100 MHz, DMSO-d_6), δ (ppm): 170.6, 151.0, 144.6, 131.5, 131.2, 123.0, 128.7, 126.1, 124.3, 124.1, 114.4, 114.1, 103.7, 59.2, 50.2, 40.5, 36.5, 34.7, 28.6, 26.0. HRMS (MALDI-TOF): *m/z* 706.1880 (M^+, calcd. 706.1890).

References

1. J. Zhang, M. Yang, C. Li, N. Dorh, F. Xie, F.-T. Luo, A. Tiwari and H. Liu, *J. Mater. Chem. B*, 2015, **3**, 2173-2184.
2. R. C. Brown, Z. Li, A. J. Rutter, X. Mu, O. H. Weeks, K. Smith and I. Weeks, *Org. Biomol. Chem.*, 2009, **7**, 386-394.
